# Sequential removal of oppositely charged multi pollutants from wastewater using sugarcane bagasse

**DOI:** 10.1038/s41598-026-62305-9

**Published:** 2026-07-22

**Authors:** Magda A. Akl, Azza A. Fahim, Aya G. Mostafa

**Affiliations:** Department of Chemistry, Faculty of Science, University of Mansoura, Mansoura, 31556 Egypt

**Keywords:** Sequential adsorption, Alizarin red S, Crystal violet, Methyl green, Sugarcane bagasse, Chemistry, Environmental sciences, Materials science

## Abstract

Efficient removal of organic pollutants from water remains a major challenge. In this work, a circular economy strategy is adopted to address critical challenges associated with excessive waste generation, resource limitations, and economic sustainability. Sugarcane bagasse (SCB), an abundant lignocellulosic agricultural waste, was utilized as a sustainable adsorbent for the sequential removal of oppositely charged dyes from aqueous solutions. Pristine SCB was first employed for the adsorption of the anionic dye Alizarin Red S (ARS), and the resulting spent adsorbent (SCB@ARS) was subsequently reused without regeneration for the removal of the cationic dyes Crystal Violet (CV) and Methyl Green (MG). The adsorbents were characterized using point of zero charge (pH_PZC_), Fourier-transform infrared spectroscopy (FTIR), Thermogravimetric analysis (TGA), Scanning electron microscopy (SEM), and Energy-dispersive X-ray spectroscopy (EDX). The effects of adsorption parameters, including shaking time, pH, dye concentration, and adsorbent dose, were studied. The maximum adsorption capacities of SCB for ARS and SCB@ARS for CV and MG were 99.38, 136.84, and 452.26 mg/g, respectively. Kinetic, isotherm, and thermodynamic studies indicated that the adsorption process was best described by the linear pseudo-2nd-order and nonlinear pseudo-1st-order kinetic models and the Langmuir isotherm model, and was thermodynamically feasible and spontaneous. The adsorption mechanism was further elucidated using pH_PZC_ and FTIR analyses. Recovery studies in pharmaceutical and real water samples demonstrated removal efficiencies exceeding 90%. A one-way ANOVA statistical analysis was used to evaluate the effect of pH on adsorption. These findings highlight the potential of sequential adsorption using renewable agricultural waste as an effective and sustainable wastewater treatment strategy.

**Supplementary Information:**

The online version contains supplementary material available at 10.1038/s41598-026-62305-9.

## Introduction

Water is a fundamental resource for all life forms. Water contamination is recognized as one of the most significant global challenges in recent decades. This issue is primarily attributed to the depletion of water resources, rapid industrialization, and substantial population growth. Contamination arises from increased concentrations of pollutants, including toxic heavy metal ions, inorganic anions, micropollutants, and organic compounds such as dyes, phenols, pesticides, and detergents^1–3^.

Synthetic dyes are among the most hazardous organic sources of water contamination and may be classified as cationic, anionic, or non-ionic^4^. Due to their high stability against light, heat, and oxidizing agents, as well as their non-biodegradable nature, these dyes exhibit significant toxicity to aquatic systems^5^. Anthraquinone and azo dyes are the two main types of organic dyes. Textiles, leather, paints, paper, plastics, and cosmetics are just a few industries in which they can be used^6^.

Alizarin red sulfonate dye (ARS) is a typical dihydroxyanthraquinone soluble in water^7,8^. It is used to identify calcium in tissue sections as a histochemical stain for bone tissue. ARS dye changes its color and λ_max_ with pH^9^.

Crystal violet dye (CV) is a dark green powder, slightly soluble in water, that belongs to the class of triphenylmethane dyes. In medical applications, CV is the active component of Gram’s stain, and it is commonly used as a bacteriostatic agent and an external skin disinfectant for humans and animals^10^. CV is also widely used in industry as a purple dye for cotton and silk textiles, as well as in paints and printing inks. The toxicity of this dye is attributed to its easy penetration into animal cells via interactions with negatively charged components of mammalian cell membranes^11^.

Methyl Green (MG) is a crystalline powder ranging from dark red to brown and is a triarylmethane. The colorant is easily soluble in ethanol and highly soluble in water. The λ_max_ of MG occurs at 630–634 nm. MG is distinguished from crystal violet by containing seven methyl groups rather than six. The seventh group can be easily lost, leading to the dye reverting to crystal violet. MG dye is utilized in staining solutions for medical and biological purposes^12^.

Internationally, there are no established specific permissible limits for either ARS or MG in environmental or agricultural/food samples. However, these dyes are classified as toxic and have a high potential for ecotoxicity, and should not be discharged into water bodies. For CV, the European Union (EU) set a permissible residue limit for fish, shrimp, and aquaculture products of not more than 0.5 µg/L^13,14^.

Exposure to water containing residual amounts of these dyes has been associated with various adverse health effects, including skin and eye irritation, digestive system disorders, respiratory complications, and an increased risk of cancer^14–18^.

These dyes must be removed from contaminated water immediately in order to address the serious problem mentioned above. A wide range of physical and chemical treatment technologies has been investigated for removing dyes from wastewater. Chemical precipitation, coagulation and flocculation, ion exchange, liquid–liquid extraction, physical flotation, cloud point extraction, and adsorption are treatment methods developed to eliminate dyes from aqueous solutions^19–22^. The advantages and disadvantages of some of these techniques are listed in Table 1.

**Table 1:** The advantages and disadvantages of water treatment techniques.

Technique	Advantages	Disadvantages	References
Chemical precipitation	Low cost Ease of operation Suitable for automatic operation control Can operate at high flow rates Can treat wastewater with high concentrations of toxic metals	Large amounts of chemicals A large amount of hazardous sludge The high cost and long-term environmental impacts of sludge disposal Low efficiency for low metal concentrations	^52^
Ion exchange	No loss of adsorbent on regeneration Reclamation of solvent after use The removal of soluble dyes Little energy consumption No sludge generation	High cost of synthetic resins Rapid exhaustion of the resin Regeneration of the resins can cause serious secondary pollution The ion exchange method is not very effective for disperse dyes	^53^
Coagulation and flocculation	Cost–effective Simple Efficient Does not release toxic substances	High consumption of chemicals Large production of hazardous sludge Must be followed by other treatment techniques	^54–56^
liquid–liquid extraction	Simple operation Simple apparatus	Need to use large volumes of solvents of high purity, which can increase environmental pollution Often, a small enrichment factor for the analyte is used Low selectivity Formation of emulsions that are difficult to break Problems with handling samples of large volume	^57^
Adsorption	Cost effective Easy to operate Highly efficient High adsorption rates Availability of a wide selection of adsorbents Absence of chemical sludge	Low selectivity	^58^

In recent years, adsorption has been considered a more desirable technique for removing dyes among the other methods mentioned. It has ease of use, non-toxicity, high efficiency, affordability, readily available adsorbents, and the capacity to manage large-scale systems^23^. Therefore, a crucial challenge in the adsorption process is the development of effective, readily available adsorbents.

Agricultural waste-derived adsorbents, specifically lignocellulosic materials containing cellulose, hemicellulose, and lignin, provide various functional groups such as hydroxyl (-OH), amino (-NH_2_), carboxyl (-COOH), phosphate (PO_4_^3-^), and carbonyl groups (-C = O), which act as active binding sites for pollutants^24^. These adsorbents possess distinct characteristics, such as a porous structure, extensive surface area, pore volume, varied pore-size distribution, ease of separation, reusability, chemical stability, affordability, abundant availability, and eco-friendliness, making them ideal for remediating dyes as organic pollutants^25,26^. On the other hand, if a large volume of these wastes produced by different agricultural sectors is not managed properly, it could result in significant health hazards and environmental issues, including food poisoning, poor food hygiene, and contamination of agricultural products and groundwater^27^.

Different, low-cost adsorbents from a variety of agricultural wastes have been utilized, including rice husk^28^, peach stones^29^, almond shells^30^, banana peels^31^, sugarcane bagasse^32^, tea waste^33^, maize cobs^34^, olive stones^35^, cherry stones^36^, and peanut shells^37^.

Sugarcane bagasse (SCB) is a heterogeneous fibrous residue that contains 40–50% cellulose, 25–35% hemicellulose, and 20–30% lignin, so it reacts actively with dyes and heavy metals^38^. The primary constituent in SCB is cellulose. It is a natural linear polymer consisting of anhydroglucose units joined by β-1,4 glycosidic bonds. It has three hydroxyl groups with varying reactivity: a primary -OH at C-6, and secondary -OH groups at C-2 and C-3^39^. These hydroxyl groups contribute to the formation of strong intramolecular and intermolecular hydrogen bonding, making it an effective adsorbent for the removal of dyes^40^.

The strategic utilization of SCB as an agricultural waste-derived adsorbent directly aligns with and supports multiple United Nations Sustainable Development Goals (SDGs). Primarily, it advances SDG 3 (Good Health and Well-being) by removing toxic, mutagenic, and potentially carcinogenic dyes from aquatic environments, minimizing human health risks. It also addresses SDG 6 (Clean Water and Sanitation) by ensuring efficient wastewater remediation and improving water quality. Furthermore, transforming this abundant byproduct into a valuable adsorbent promotes SDG 12 (Responsible Consumption and Production) by minimizing waste and advancing circular economy principles. Additionally, converting agricultural waste into eco-friendly products reduces carbon emissions, contributing to SDG 13 (Climate Action). Moreover, it supports SDG 14 (Life Below Water) by preventing the discharge of chemical pollutants into receiving water bodies, thereby safeguarding the marine environment and preserving aquatic ecosystems^41–45^.

Numerous studies have utilized SCB-derived adsorbents for the removal of various pollutants. Recently, Akl et al. combined SCB-activated carbon with Fe_3_O_4_ magnetic particles to achieve efficient and rapid separation of Pb(II) and Hg(II) from diverse water samples^46^. Additionally, a chitosan-impregnated sugarcane bagasse biochar (SCNC) biocomposite was developed for the removal of anionic Congo red dye from aqueous solutions^47^. Sulfonated SCB has also been employed for the removal of cationic methylene blue and Bismarck brown R dyes^48^.

Sequential dye adsorption enhances selectivity by controlling the adsorbent surface charge and more accurately simulates real wastewater containing multiple charged pollutants^49–51^.

The novelty of the current investigation is not limited to the adsorption of ARS onto the SCB adsorbent or to the SCB@ARS preparation; but also, it lies in the development of an oppositely charged sequential adsorption approach. The current investigation presents adsorption of the anionic dye ARS as a deliberate surface-modification step to enhance the subsequent removal of the cationic dyes, CV and MG. In contrast to conventional adsorption investigations focusing on single-pollutant (adsorbate) removal and on the simultaneous adsorption of mixed solutions, the current investigation employs a sequential technique for the adsorption of differently charged pollutants. When the ARS was adsorbed onto the SCB surface, negatively charged SO_3_^-^ groups were introduced, altering surface chemistry. This results in an enhanced affinity toward cationic species (CV and MG). The current study introduces a sustainable, affordable, and simple approach for producing a functional adsorbent from low-cost agro-waste that can treat water effluents containing differently charged pollutants.

This investigation demonstrates several advantages, such as environmental sustainability, cost-effectiveness, operational simplicity, waste valorization, and scalability. Furthermore, the introduction of functional groups during the initial adsorption step of ARS (in situ surface modification) facilitates the removal of a secondary pollutant with an opposite charge (CV and MG) and reduces reliance on toxic chemicals. Compared with previously reported adsorption studies, which mainly investigated single-dye removal, simultaneous adsorption from mixed solutions, or the preparation of chemically modified adsorbents using additional reagents, the present study introduces a different sequential adsorption concept. The current investigation is based on adsorbate-induced surface functionalization. Although a limited number of studies have explored multi-step or sequential removal of pollutants, the underlying mechanisms differ from the present approach. For instance, Tamer et al.^49^ removed the negatively charged Cr_2_O_7_^--^ using immobilized Crystal violet on nano-sulfonated Poly(glycidyl methacrylate) nanocomposite, in which surface modification was performed prior to adsorption rather than generated in situ by the first pollutant. Therefore, the novelty of this work lies in utilizing the first adsorbed pollutant as an active modifier to regulate surface charge and enable enhanced sequential adsorption of oppositely charged contaminants.

The ARS anionic azo dye was immobilized onto SCB particles via adsorption in the current work, yielding a new adsorbent (SCB@ARS) that demonstrated affinity for removing the cationic dyes MG and CV from contaminated water samples.

The objectives of this study are:

1. Adsorption of ARS onto SCB to yield SCB@ARS, which was reutilized for sequential removal of the cationic dyes viz. CV and MG.
2. Characterization of SCB and SCB@ARS using different instrumental performances, such as BET, SEM, EDX, TGA and FT-IR.
3. Optimize ARS removal using SCB through batch adsorption investigations and studying several parameters, including dose, pH, initial concentration, and oscillation time.
4. Experimental investigation of cationic pollutants CV and MG adsorption on the surface of SCB@ARS by investigating several parameters, such as adsorbent dosage, pH, initial concentration of CV, and MG concentrations, and ionic strength.
5. Conducting adsorption isotherm and kinetic experiments to understand the adsorption mechanism and the highest adsorption performance of SCB@ARS.
6. Application of the prepared SCB@ARS biosorbent on real water samples.
7. Studying the adsorption of CV and MG cationic species in multi-contaminant systems.
8. Elucidation of the adsorption mechanism of ARS onto SCB and the CV and MG onto SCB@ARS biosorbent.

## Experimental

### Materials and method

#### Materials

The pristine SCB waste was collected from the local market, Mansoura, Egypt. Alizarin red S (Merck), crystal violet (Sigma-Aldrich), and methyl green (Merck) were used. HCl (Merck, 98%) and NaOH (Sigma-Aldrich, Lab grade) were used. The chemical structures of ARS, CV, and MG are presented in Table S1.

#### Preparations

##### Preparation of pristine SCB biosorbent

Preparation of pristine SCB was performed as previously reported^59^. The pristine SCB waste was collected, cut, and ground well to the desired size in microscale (< 500µm). The obtained powder was then stirred into distilled water, filtered, and air-dried.

##### Preparation of SCB@ARS biosorbent

The SCB@ARS biosorbent was obtained via batch adsorption experiments with ARS under optimal conditions: 0.5 g/L SCB and 50 mg/L ARS at pH 2 for 120 min at room temperature. Once the adsorption process was completed, the SCB@ARS biosorbent was filtered and washed to remove any excess ARS. The SCB@ARS biosorbent was used for the subsequent adsorption of the cationic CV and MG dyes from polluted water samples.

### Instrumentation

Fourier transform infrared (FTIR) spectra of pristine SCB, SCB@ARS, SCB@ARS@CV, and SCB@ARS@MG were recorded over the wavenumber range of 4000–400 cm^–1^ using a PerkinElmer Spectrum RX I with KBr pellets. The surface morphology of SCB, SCB@ARS, and SCB@ARS@CV was investigated using a JEOL JSM-6510LV scanning electron microscope, which was also used to obtain EDX (Energy-dispersive X-ray Spectroscopy) spectra. The thermal stability of SCB and SCB@ARS materials was examined by thermogravimetric analysis (Berkin Elmer TGA 4000) at a heating rate of 15 °C/min from 30 to 900 °C. The specific surface area of S_BET_, pore volume, and pore diameter of SCB and SCB@ARS were estimated using the Brunauer Emmett Teller, BET, analysis (MicrotracBEL Corp., Japan) operated through BELMaster software (Version 7.3.2.0). Prior to measurements, the samples were degassed under vacuum at 80 °C for 3 h to remove physically adsorbed impurities and moisture. A Perkin-Elmer 550 spectrophotometer was used to determine the concentrations of the investigated dyes (ARS, CV, and MG) and the CV-MG binary system over a wavelength range of 200–900 nm with quartz cells. The maximum absorption wavelengths (λ_max_) were identified as 412 nm for ARS, 590 nm for CV, 645 nm for MG, and 629 nm for the CV-MG binary system. The pH_pzc_ of SCB and SCB@ARS were determined as previously reported^60^.

### Batch adsorption tests

Batch adsorption experiments were performed to investigate the adsorption capacity of SCB towards ARS. Then, SCB loaded with ARS (SCB@ARS) was used for the removal of cationic dyes, used as secondary pollutants (CV& MG).

#### ARS adsorption tests

The tests were carried out in 50 mL bottles, maintaining a fixed adsorbent dosage (0.5 g/L) and varying the ARS concentration (10–150 mg/L) of 10 mL of solution at pH 2. The solutions were shaken (150 rpm) at room temperature for 120 min. Finally, SCB was removed by centrifugation, and the resulting solutions were analyzed by UV–vis spectroscopy (Shimadzu, UV-2600) at λ = 412 nm . Various effects, including pH levels between 2 and 12, SCB doses from 0.0025 to 0.02 g, temperature ranging from 25 °C to 45 °C, and oscillation durations from 15 to 180 min, were studied for the adsorption of ARS dye onto the surface of SCB. The removal percentage (%) and the equilibrium adsorption capacity (q_e_) were calculated using the Eqs. (1) and (2), respectively. Each experiment was carefully repeated three times to ensure consistent results.

1 $$\mathrm{R}\left(\mathrm{\%}\right)=\frac{{\mathrm{C}}_{\mathrm{i}}-{\mathrm{C}}_{\mathrm{f}}}{{\mathrm{C}}_{\mathrm{f}}}\times 100$$

2 $${ \mathrm{q}}_{\mathrm{e}}=\frac{\left({\mathrm{C}}_{\mathrm{i}}-{\mathrm{C}}_{\mathrm{f}}\right)\times \mathrm{v}}{\mathrm{m}}$$

C_i_ and C_f_ represent the starting and final concentration values in mg/L, respectively. while m (g) represents the adsorbent dose, and V(L) represents the volume of the adsorbate solution.

#### Cationic dyes batch adsorption tests

SCB used for the removal of ARS (now SCB@ARS) was subsequently tested for the removal of cationic dyes (CV and MG) as secondary pollutants. In more detail, 5 mg of SCB@ARS were added to 10 mL of 150 mg/L and 250 mg/L of CV and MG dye solutions, respectively. Each mixture was stirred for 120 min at room temperature. Aliquots were sampled, and the dye concentrations were verified by UV–vis spectroscopy at λ_max_ = 590 nm, λ_max_ = 645 nm, for CV, MG, respectively. The effects of solution pH (3–11), initial dye concentration (50–300 mg/L), adsorbent dosage (0.0025–0.02 g), solution temperature (25–45^○^C), and contact time (15–180 min) were examined. Adsorption efficiency was expressed in terms of removal percentage (%) and equilibrium adsorption capacity (q_e_) using Eqs. (1) and (2). Each experiment was carefully repeated three times to ensure consistent results.

**Fig. 1:**
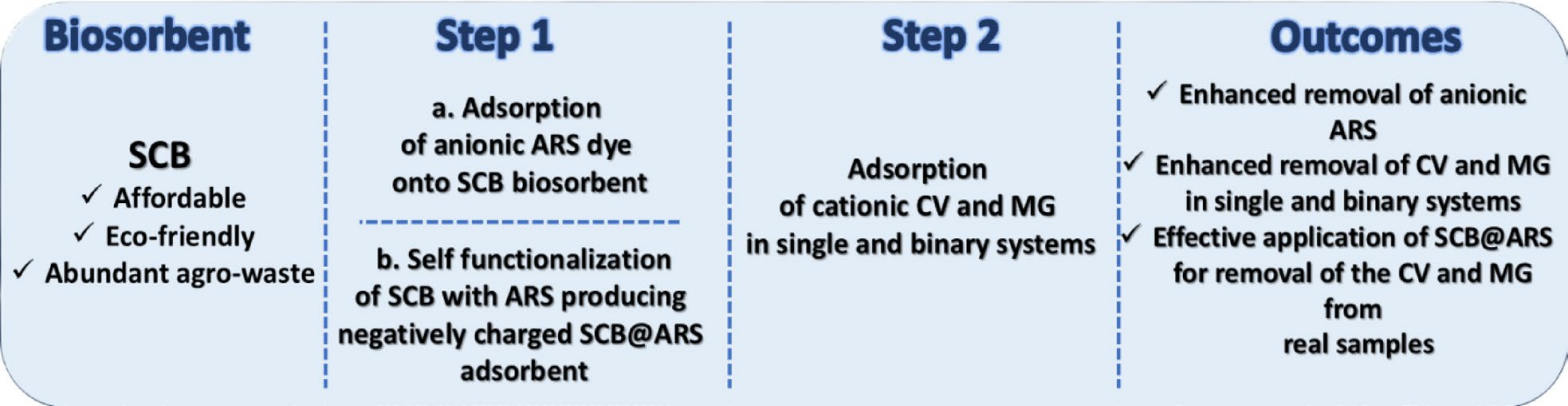
Schematic illustration of the adsorption of ARS onto SCB and sequential adsorption of CV and MG onto SCB@ARS.

Figure 1 illustrates the main steps and outcomes of the current investigation.

The detailed kinetic, isotherm, thermodynamic analyses, and error function calculations are provided in the Supporting Information.

#### Removal of CV and MG in a binary system

To assess SCB@ARS efficiency as an adsorbent in a binary dye system (CV–MG), batch adsorption experiments were conducted. A 0.01 g sample of SCB@ARS was added to 10 mL of mixed cationic dyes (150 mg/L CV, 250 mg/L MG) at pH 10. The mixture was shaken at 120 rpm and 25 °C for 30, 60, 120, and 180 min. Equilibrium concentrations were determined via UV–Vis data, and adsorption capacity was calculated using Eq. (2).

#### Leaching stability test

The SCB@ARS adsorbent was prepared by adsorbing ARS onto pristine SCB at pH 2. The stability of adsorbed ARS on SCB@ARS was evaluated through a leaching study conducted at various pH levels. In this experiment, 0.005 g of SCB@ARS was evaluated in 10 mL solutions with pH values ranging from 2 to 10 for 48 h at room temperature. The solution was measured using a Unicam UV-2100 UV/Visible spectrophotometer at 412 nm (pH 2), 425 nm (pH 4), 515 nm (pH 6), and 550 nm (pH 8–10).

#### Applications in real samples

##### Natural water samples

To evaluate the applicability of SCB@ARS for the removal of the studied dyes from real water samples, water samples were collected from several sources, including Misr Spinning and Weaving Company in El-Mahalla El-Kubra (before and after treatment), seawater from Port Said, and laboratory tap water at Mansoura University. The collected samples were spiked with (50–150) mg/L and (50–250) mg/L of CV and MG, respectively.

Prior to spiking with the dyes, the natural water samples were digested by adding 0.5 g of K₂S₂O₈ and 5 mL of H₂SO₄ (98% w/w) to 1000 mL of the water sample, followed by heating at 90 °C for 120 min to ensure complete digestion of organic matter^61^. After cooling to room temperature, 0.005 g of SCB@ARS was added for CV removal and 0.01 g for MG removal. The samples were then continuously shaken for 120 min at pH 10.

The residual concentrations of CV and MG were determined using a Unicam UV-2100 UV/Visible spectrophotometer at wavelengths of 590 nm and 645 nm for CV and MG, respectively.

##### In pharmaceutical samples

Gentian Violet (0.5%, 5000 mg/L), purchased from a commercial pharmacy, was used as a model pharmaceutical sample to evaluate the adsorption performance of SCB@ARS. Gentian violet belongs to the group of medicines called antifungals. Topical gentian violet is used to treat some types of fungal infections inside the mouth (thrush) and on the skin. To investigate the applicability of the SCB@ARS adsorbent, a range of (50–150) mg/L Gentian violet solution at pH 10 was mixed with 0.01 g of SCB@ARS for 120 min. A Unicam UV-2100 UV–visible spectrometer was used to measure the remaining amounts of CV.

## Results and discussion

### Digital photographs

**Fig. 2:**
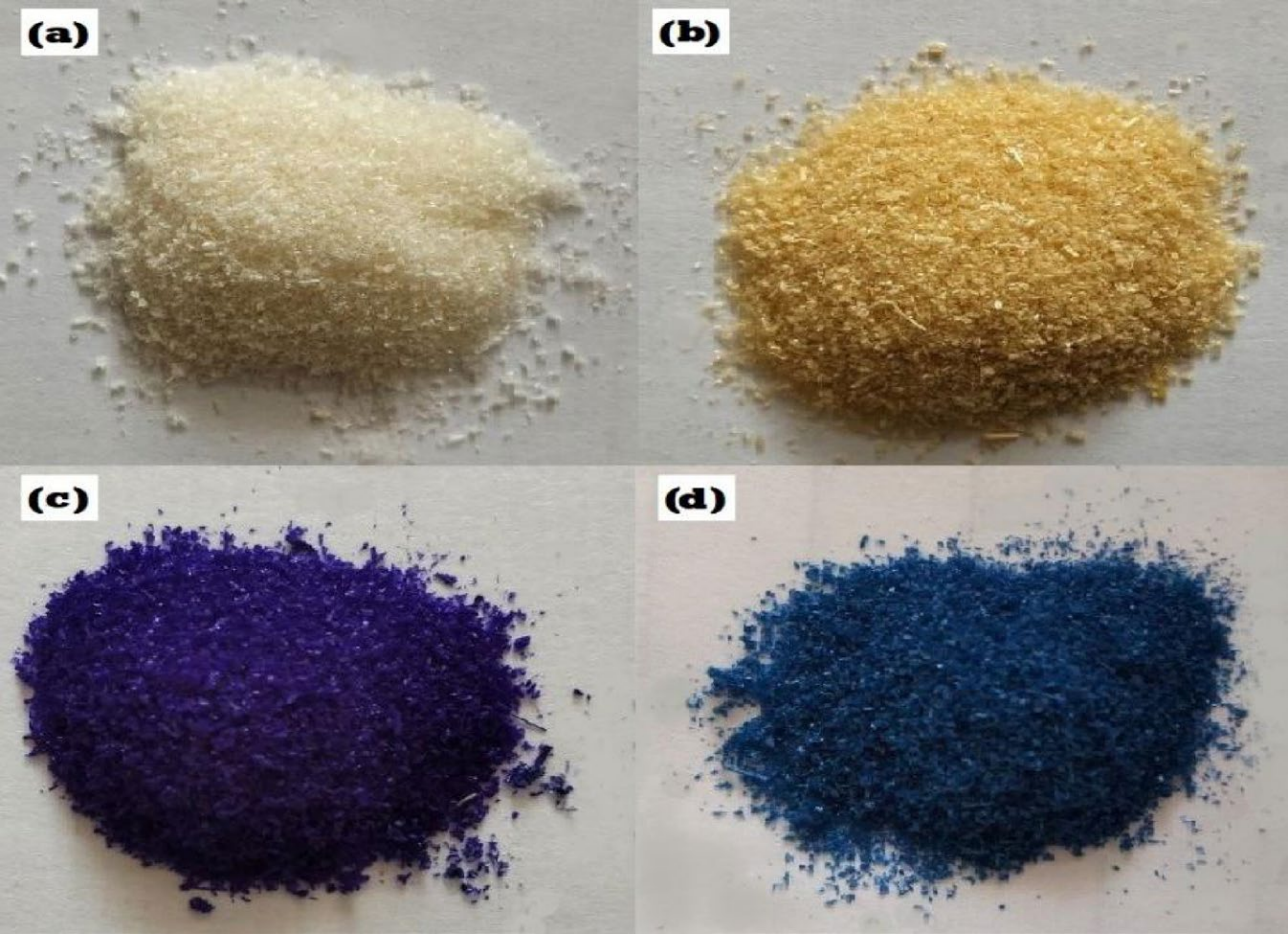
Digital photographs of **(a)** pristine SCB, **(b)** SCB@ARS, **(c)** SCB@ARS@CV, **(d)** SCB@ARS@MG.

Figure 2a-d presents digital photographs of pristine SCB, SCB@ARS, SCB@ARS@CV, and SCB@ARS@MG. The pristine SCB appears off-white **(**Fig. 2a). Upon reaction with ARS dye, the color changes to golden yellow (Fig. 2b), indicating ARS dye adsorption. Building on this, subsequent adsorption of CV and MG dyes onto SCB@ARS biosorbent, shifts the color further: to violet (Fig. 2c) after CV adsorption, and to green (Fig. 2d) following MG adsorption. These sequential color changes clearly demonstrate each adsorption step on the SCB@ARS surface.

### Characterization

#### BET

The specific surface area of the adsorbent (S_BET_) is a critical parameter in adsorption, as it directly determines the adsorbent’s capacity to remove dyes^62^. Textural characteristics, including S_BET_, pore volume (cm^3^/g), and average pore diameter (nm) for SCB and SCB@ARS, were measured and are presented in Table 2 and Fig. S1. The S_BET_ of SCB decreased from 3.98 m^2^/g to 1.28 m^2^/g after ARS adsorption, accompanied by a reduction in pore volume (cm^3^/g) to 1.04 × 10^–2^ cm^3^/g. This reduction may be attributed to the SCB surface being partially covered by ARS particles, which limit N_2_ accessibility. In contrast, the average pore diameter increased after ARS adsorption, likely due to the blockage of smaller pores and the relative contribution of larger pores. Both SCB and SCB@ARS exhibit pore diameters within the mesoporous range. However, given the relatively low BET surface area, the adsorption enhancement observed in this study cannot be attributed solely to porous characteristics, as the pores are larger than those of ARS (1–2 nm), CV (1.3–1.5 nm), and MG (1.5–1.8 nm). Which may facilitate ARS diffusion through SCB, as well as CV and MG diffusion through SCB@ARS. The mesoporous structure may contribute to adsorbate accessibility, while surface chemical interactions dominate the adsorption process.

**Table 2:** Textural characteristics of SCB and SCB@ARS biosorbents.

Sample	S_BET_ (m^2^/g)	Pore volume (cm^3^/g)	Average pore diameter (nm)
SCB	3.98	2.198 × 10^–2^	22.096
SCB@ARS	1.28	1.04 × 10^–2^	32.608

#### EDX

**Fig. 3:**
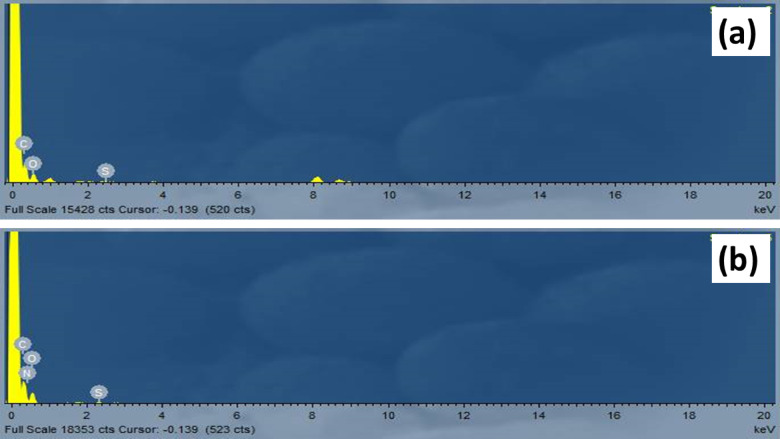
EDX of **(a)** SCB@ARS biosorbent and **(b)** SCB@ARS@CV.

Figure 3 displays the EDX spectra for SCB@ARS biosorbent and SCB@ARS@CV samples. EDX analysis was conducted to determine the elemental composition of the materials. The EDX spectrum of pristine SCB shows 57.37% carbon and 42.36% oxygen, as previously reported^48^. After ARS adsorption, the EDX spectrum of SCB@ARS (Fig. 3a) exhibits a sulfur peak, confirming ARS adsorption onto the SCB surface. Subsequently, following CV adsorption, the EDX spectrum of SCB@ARS@CV (Fig. 3b) reveals a nitrogen peak, indicating substantial adsorption of CV, which contains a high nitrogen content.

#### FT-IR spectra

**Fig. 4:**
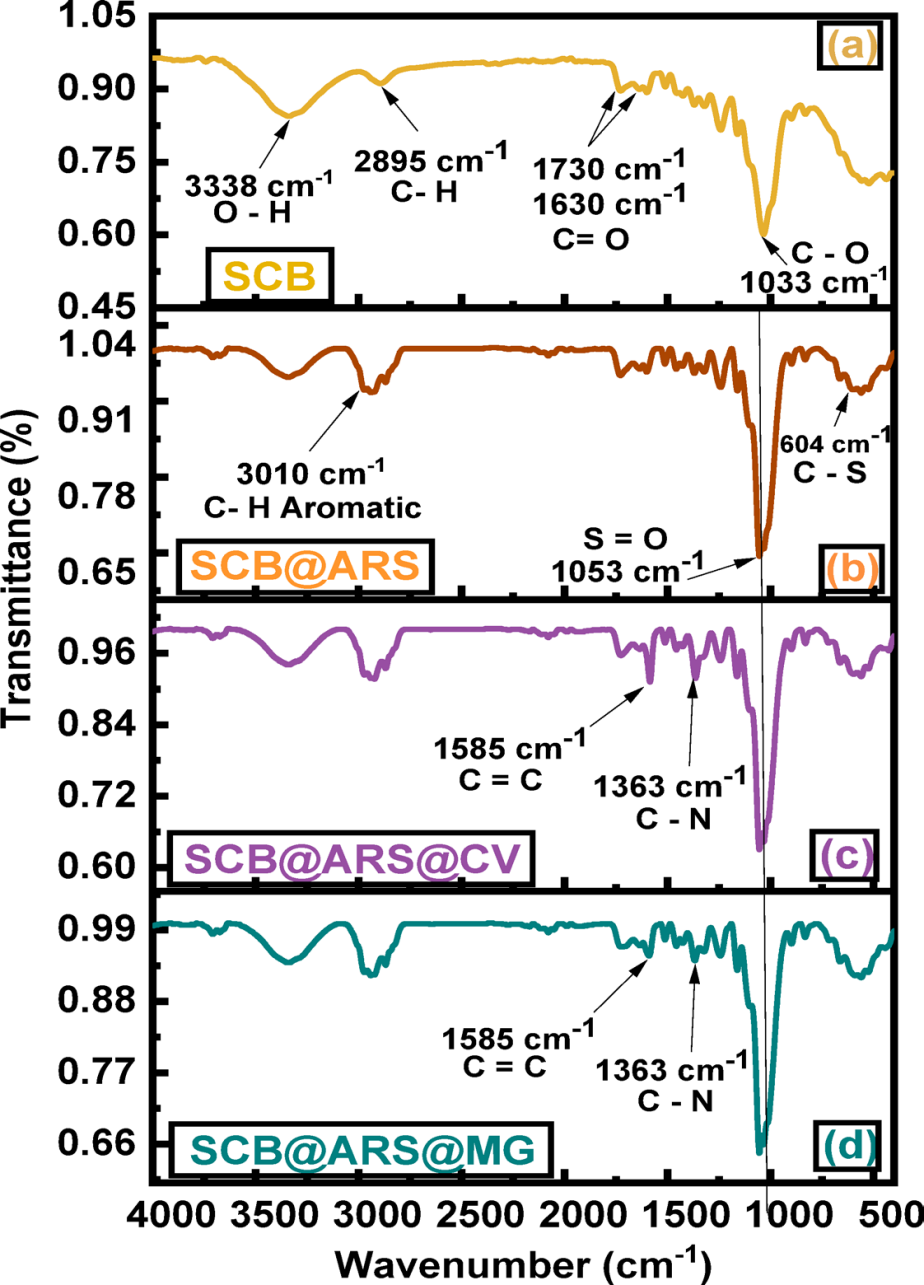
FT-IR spectra of **(a)** SCB, **(b)** SCB@ARS, **(c)** SCB@ARS@CV, and **(d)** SCB@ARS@MG.

FTIR spectra of unmodified sugarcane bagasse (SCB), SCB@ARS, SCB@ARS@CV, and SCB@ARS@MG are shown in Fig. 4.

FT-IR spectra for SCB showed characteristic bands at 3338 cm^−1^ corresponding to the stretching vibration of hydroxyl functional groups^63^, 2895 cm^−1^ related to C–H stretching^64^, peaks at around 1730 and 1630 cm^−1^ can be assigned to C = O vibrations in hemicellulose and lignin^65,66^, and finally 1033 cm^−1^ because of C–O^67^.

The FT-IR spectrum of SCB@ARS shows distinct changes, including a noticeable increase in the width of the OH stretching band, indicating stronger hydrogen-bonding interactions. A strong band at 1053 cm^–1^ corresponds to the S = O stretching vibration from the sulfonate group found in ARS^68^, while another band at 604 cm^–1^ pertains to C-S stretching^69^. The spectrum also shows distinct aromatic characteristics originating from the ARS dye, with two new peaks seen at 1925 cm^–1^ and 3010 cm^–1^ associated with stretching vibrations of aromatic C-H groups^70^. Moreover, the newly observed peak at 716 cm^–1^ is linked to the out-of-plane bending of C-H bonds within the aromatic ring frameworks^71^. Lastly, the higher intensity of the 1456 cm^–1^ band is associated with the in-plane bending of C-H bonds^72^. These findings validate that ARS dye was effectively adsorbed onto the surface of SCB material, evidenced by the new sulfonic and aromatic signals absent in the spectrum of the unmodified sample. In the FT-IR spectrum of SCB@ARS@CV and SCB@ARS@MG, the peak at 1585 cm^–1^ became more intense, indicating the presence of C = C bonds of the alkene of the aromatic structure^73^, which can be attributed to the rising quantity of aromatic rings formed from the interaction between SCB@ARS and the two dyes. Additionally, the intensity of the peak at 1363 cm^–1^ increased because of the C-N bond^74^ present in the two dyes’ structure.

#### SEM

**Fig. 5:**
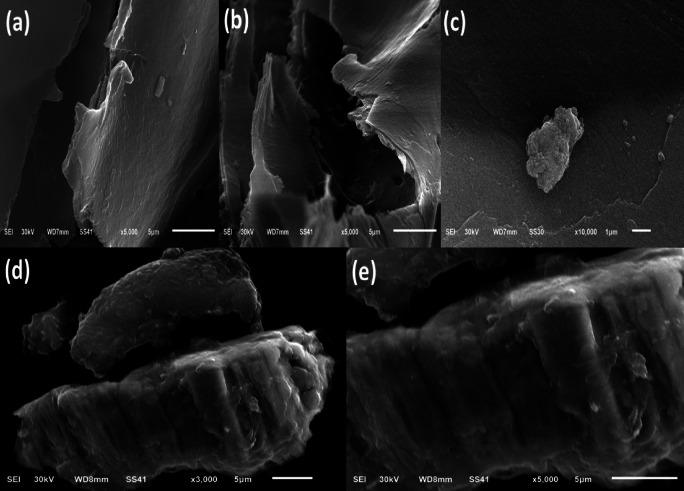
SEM images of: **(a)** pristine SCB at 5000x **(b)** SCB@ARS at 5000x **(c)** SCB@ARS at 10000x, **(d)** SCB@ARS@CV at 3000x, and **(e)** SCB@ARS@CV at 5000x.

The surface morphology of raw SCB, SCB@ARS, and SCB@ARS@CV was investigated by SEM and presented in Fig. 5a–e. As shown in Fig. 5a, the untreated SCB material exhibited a relatively smooth, compact surface with clear layered structures and visible cracks, indicating the presence of pores and active sites for dye adsorption^75^. In Figs. 5b, c, the SCB@ARS surface looked rougher than the untreated SCB. Small, uneven features appeared because the ARS dye was adsorbed to the SCB. As seen in Fig. 5d, e, the surface of SCB@ARS@CV became denser, and a more compact layer covered the surface, while the particles appeared more clustered. This indicates that the second dye (CV) was adsorbed sequentially on top of the previously adsorbed dye layer. These observations confirmed the occurrence of sequential adsorption, in which the second dye occupied the remaining or newly formed active sites on the surface after the first adsorption step.

#### TGA

**Fig. 6:**
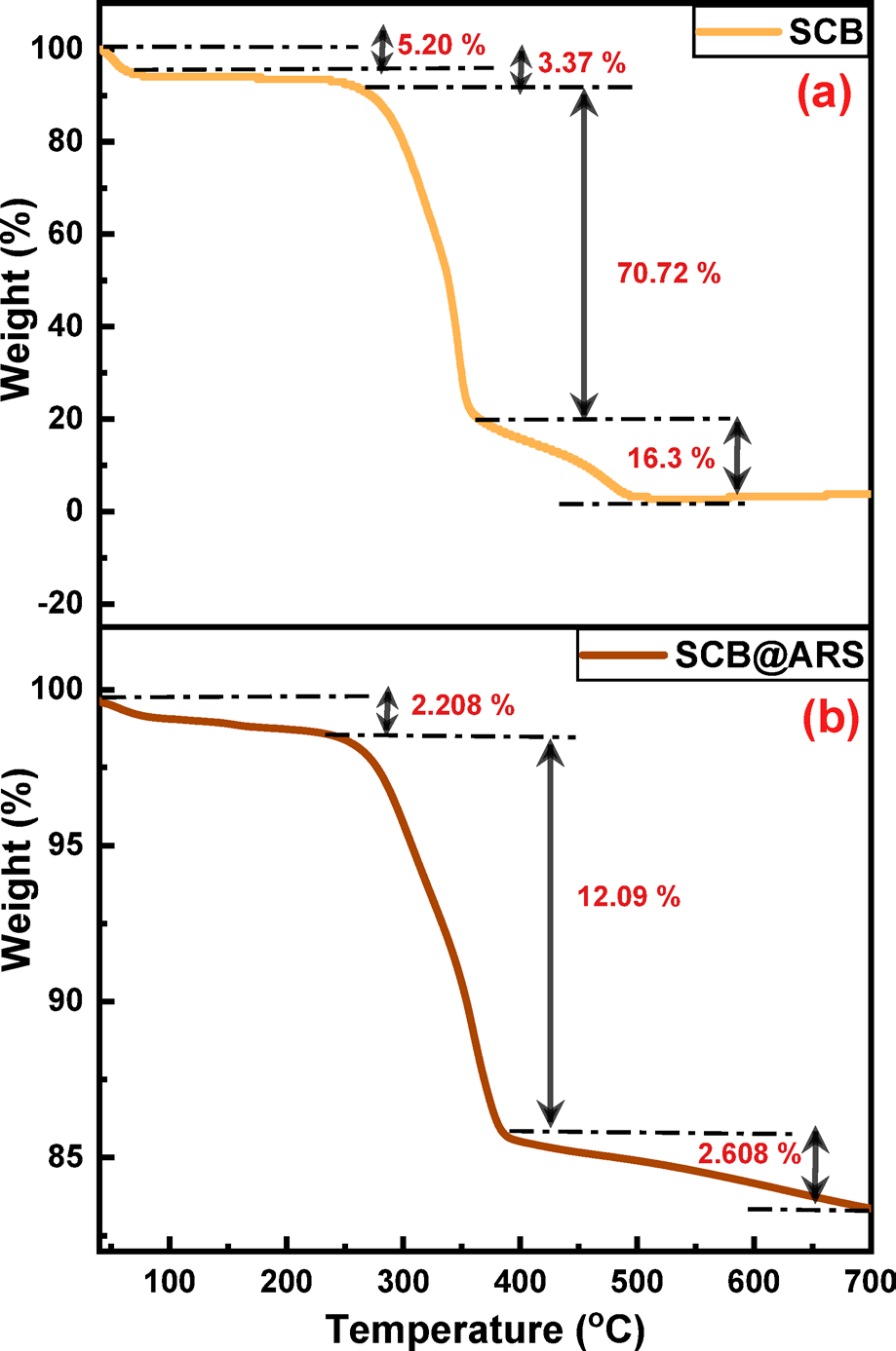
TGA of **(a)** pristine SCB and **(b)** SCB@ARS biosorbent.

The TGA patterns of the raw SCB and SCB@ARS adsorbent are shown in Fig. 6. The TGA pattern of the raw SCB (Fig. 6a) showed four main pyrolysis stages; (31–58 °C, 5.20%) due to evaporation of surface water, (58–259 °C, 3.37%) attributed to liberation of interlayer water, the main decomposition stage (259–358 °C, 70.72%) due to degradation of cellulosic chains and finally, (358– 582 °C, 16.30%) related to residue carbonization. By adsorbing ARS onto the SCB surface (Fig. 6b), the amount of absorbed surface water decreased by 2.208%. Moreover, the degradation percentage decreased from 70.72% to 12.09%, suggesting a more thermally stable structure, likely due to the strong interaction between SCB and ARS. Finally, the last stage showed a decrease in the pyrolysis percentage from 16.00 to 2.608%.

### Adsorption studies

#### Removal of ARS using pristine SCB

##### Point of zero charge (pH_PZC_) of pristine SCB

**Fig. 7:**
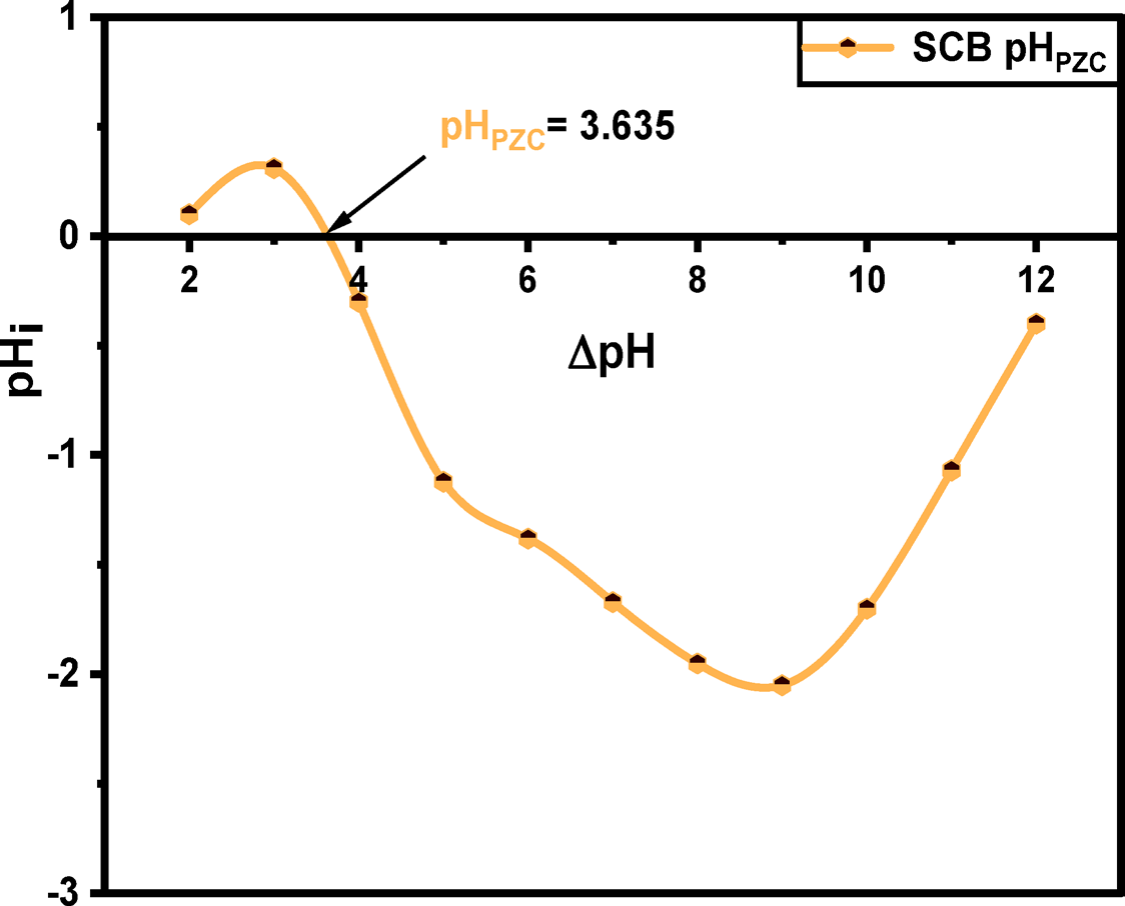
The pH_pzc_ value of the pristine SCB adsorbent.

To understand the adsorption mechanism of ARS by the SCB adsorbent, the point of zero charge (pH_pzc_) of SCB was determined. The pH_pzc_ value was found to be approximately 3.635, as shown in Fig. 7. Therefore, the adsorption of the anionic ARS dye onto SCB should be enhanced at pH values below the pH_pzc_, where the SCB surface is positively charged.

##### Effect of pH

**Fig. 8:**
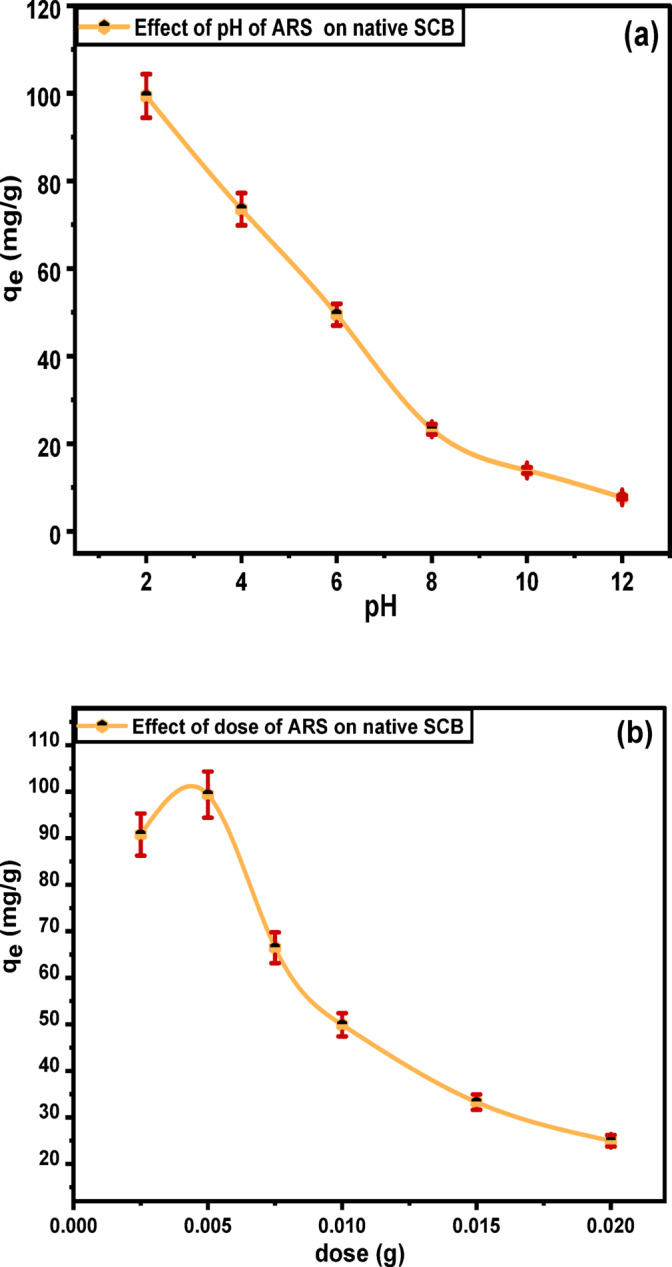
Effect of **(a)** pH (conditions: 10 mL aqueous solution of 50 mg/L of ARS and 0.005 g of SCB for 120 min) and **(b)** SCB dosage on the adsorption of ARS by pristine SCB adsorbent (conditions: 10 mL aqueous solution of 50 mg/L of ARS for 120 min at pH 2).

The effect of pH on the adsorption capacity of ARS anionic dye onto the surface of raw SCB is presented in Fig. 8a. Experiments were performed using 0.005 g of SCB in 10 mL of 50 mg/L ARS solution, with a contact time of 120 min. Results reveal significant variation in adsorption efficiency across different pH levels: equilibrium adsorption capacity (q_e_) decreases with increasing pH. Maximum adsorption was observed at pH 2 (99.38 mg/g), while minimum adsorption occurred at pH 12 (7.76 mg/g). This behavior relates to the SCB’s pH_PZC_ = 3.635. When pH is below pH_PZC_, the SCB surface is positively charged, which enhances electrostatic attraction to the anionic ARS dye. In contrast, above this point, the surface becomes negatively charged, causing electrostatic repulsion. Furthermore, at higher pH, OH^–^ ions may compete with ARS for available adsorption sites, further reducing adsorption.

##### Effect of dose

The effect of SCB adsorbent dosage was investigated over the range of 0.0025–0.02 g. The experiments were carried out at room temperature with 120 min shaking using 10 mL of a 50 mg/L solution at pH 2. As shown in Fig. 8b, increasing the SCB dose from 0.0025 to 0.005 g enhanced the removal capacity from 90.796 to 99.38 mg/g. However, when the dosage exceeded 0.005 g, the removal capacity decreased sharply, reaching 24.975 mg/g at 0.02 g. This decrease at higher dosages may be due to aggregation of adsorbent particles, which reduces the effective surface area available for adsorption. Therefore, the optimal adsorbent dosage was 0.005 g. Overall, these findings demonstrate that SCB achieves high efficiency at low dosages, emphasizing the importance of optimizing dosage to maximize removal capacity in practical applications.

##### Effect of initial ARS concentration and isotherm studies

To investigate how the initial ARS dye concentration affects its adsorption by SCB, 0.005 g of SCB adsorbent was mixed with 10 mL of ARS dye solution with concentrations ranging from 10 to 150 mg/L for 120 min at pH 2 and room temperature.

**Fig. 9:**
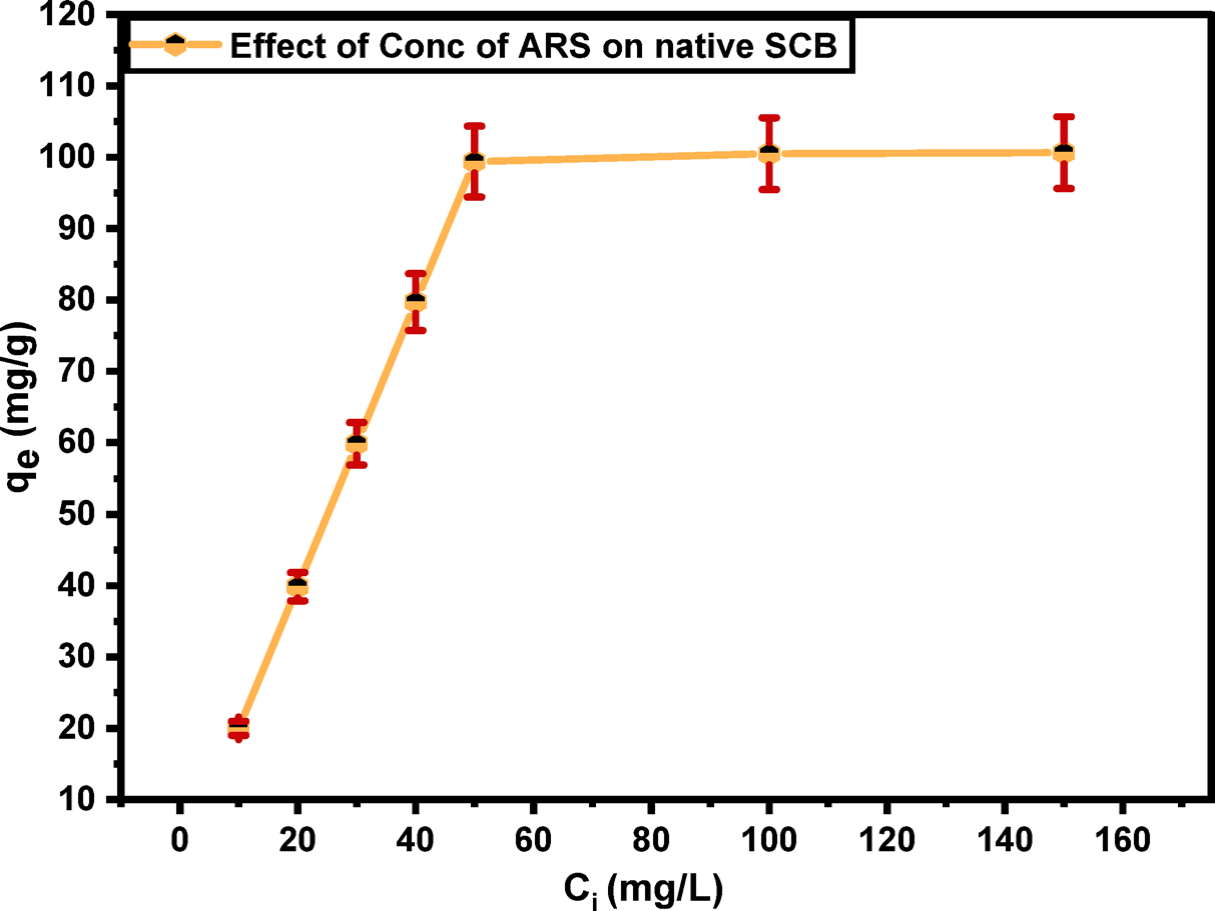
Effect of initial ARS dye concentration on its adsorption by pristine SCB (conditions: 0.005 g of SCB and shaking time of 120 min at pH 2).

Following this procedure, as shown in Fig. 9, the adsorption capacity increased from 19.99 to 99.38 mg/g as the concentration increased from 10 to 50 mg/L. Above 50 mg/L, the adsorption capacity remained nearly constant, indicating that the equilibrium was reached at this concentration.

Linear and nonlinear Langmuir, linear and nonlinear Freundlich, and Dubinin–Radushkevich (D–R) isotherm models were applied to evaluate the adsorption behavior of ARS dye onto the SCB adsorbent. The plots of the three models are presented in Fig. S2, while the calculated parameters (q_m_, K_L1_, R_L_, n2, K_F1_, K_L2_, R_L_, n2, K_F2_, and k_DR_) are listed in Table 3.

**Table 3:** Langmuir, Freundlich, and D–R isotherm constants, correlation coefficients, and error functions of ARS dye adsorption onto SCB.

Linear Langmuir isotherm constants							
	K_L_(L/g)	q_m_ (mg/g)	R^2^	R_L_	χ^2^	MSE	SSE
ARS	11.528	100.705	1	1.732 × 10^–3^	0.122	1.756	12.289

Linear Freundlich isotherm constants							
	K_F_	n	R^2^	χ^2^	MSE	SSE	
ARS	65.691	8.001	0.6588	2.063	30.925	216.473	

D–R isotherm constants							
	K	E (kJ/mol)	q_m_ (mg/g)	R^2^			
ARS	7.636 × 10^–9^	8.092	90.176	0.8606			

Nonlinear Langmuir isotherm constants							
	K_L_(L/g)	q_m_ (mg/g)	R^2^				
ARS	104.59	15.44	0.89				

Nonlinear Freundlich isotherm constants							
	K_F_	N	R^2^				
ARS	73.4	11.75	0.62				

Based on the higher correlation coefficient of the linear Langmuir model (R^2^ = 1) compared to the other applied models and the lower error function values obtained with linear Langmuir, the linear Langmuir model best fit the equilibrium data, indicating that ARS removal by SCB occurred via monolayer adsorption on a homogeneous surface. Additionally, the calculated R_L_ value (1.732 × 10^–3^) was found to be less than 1.0, indicating a favorable adsorption process and the suitability of SCB as an effective adsorbent for ARS removal.

The E_DR_ for adsorption, calculated using the Dubinin–Radushkevich model, was 8.092 kJ/mol, which falls within the 8–16 kJ/mol range, indicating a physicochemical nature with both physical and chemical interactions.

##### Effect of time

**Fig. 10:**
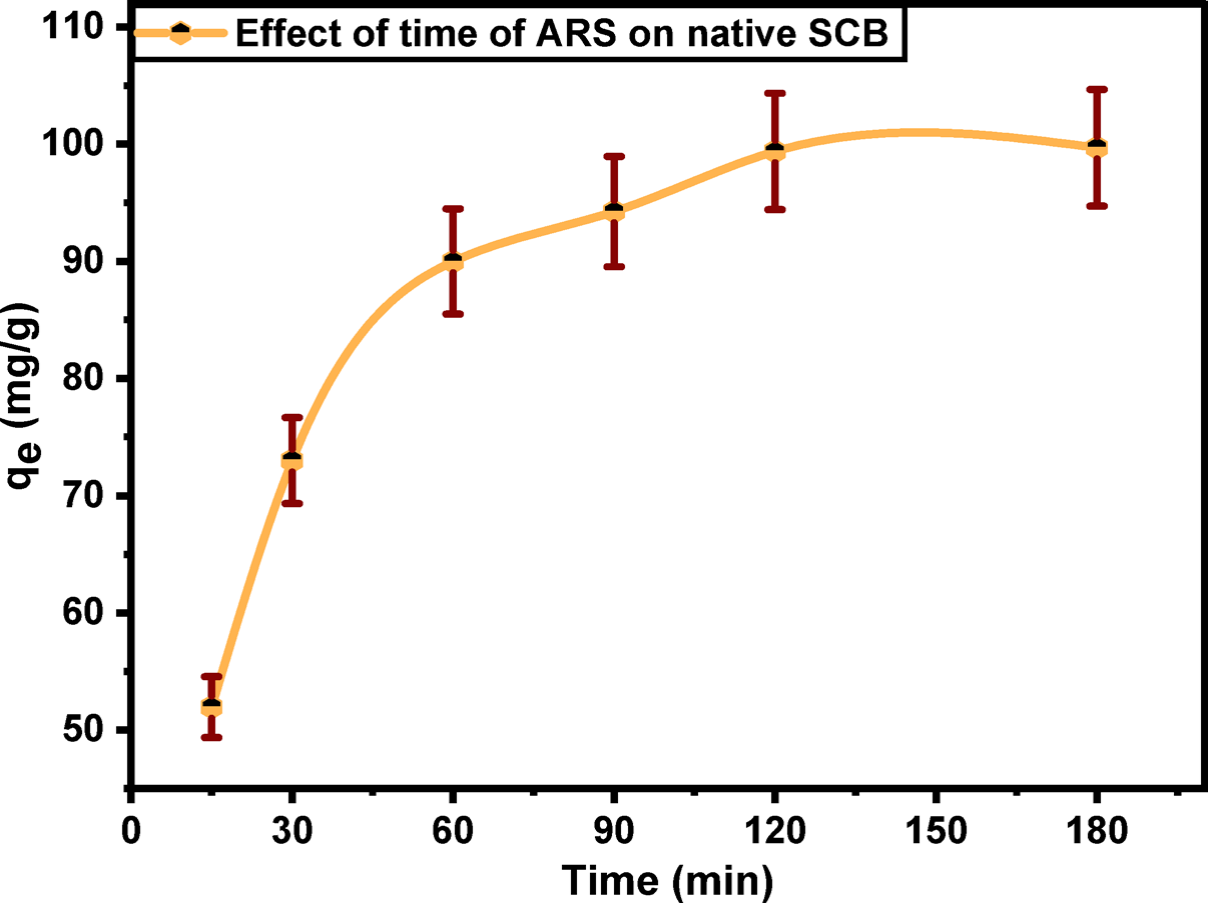
Effect of contact time on the adsorption of ARS by pristine SCB (conditions: 10 mL aqueous solution of 50 mg/L of ARS and a SCB dose of 0.005 g at pH 2).

The effect of contact time on the adsorption capacity (q_e_) of ARS dye onto pristine SCB was studied using 10 mL of a 50 mg/L ARS dye solution and 0.005 g of SCB adsorbent at pH 2. As shown in Fig. 10, q_e_ increased rapidly from 51.953 to 99.38 mg/g as the contact time increased from 15 to 120 min. This rise is due to the availability of many active sites. The maximum adsorption capacity, 99.38 mg/g, was achieved at 120 min. After this time, no significant increase in q_e_ was seen, and the curve became almost constant, indicating that equilibrium had been reached and most active adsorption sites were occupied. Thus, 120 min proved to be the ideal contact duration for ARS adsorption on pristine SCB.

The kinetics of ARS adsorption onto SCB were investigated using models: linear and nonlinear pseudo-1^st^-order, linear and nonlinear pseudo-2^nd^-order, intraparticle diffusion (IPD), and Elovich. The estimated kinetic parameters (K_1_, K_2_, K_diff_, q_e1_, and q_e2_) and the correlation coefficients (R^2^) obtained from fitting each model are summarized in Table 4. According to the R^2^ values obtained from the investigated kinetic models, the adsorption data showed better agreement with the linear pseudo-2nd-order model (R^2^ = 0.999). However, the nonlinear pseudo-1st-order model, along with the calculated q_e_ values and error functions, showed better agreement. This difference between linear and nonlinear approaches indicates that a single model cannot describe the kinetic behavior and may reflect the complex nature of ARS adsorption in SCB, involving multiple interactions and physicochemical contributions.

**Table 4:** Kinetic parameters for the adsorption of ARS by SCB.

Adsorbates	Linear- pseudo-1st order					
	q_e1_ (mg/g)	K_1_ (min^-1^)	R^2^	χ^2^	MSE	SSE
ARS	113.250	17.368	0.994	10.192	192.377	1154.261

Linear- pseudo-2nd order						
	q_e2_ (mg/g)	K_2_ (min^-1^)	R^2^	χ^2^	MSE	SSE
ARS	108.933	6.412 × 10^–4^	0.999	5.027	91.26	547.559

Nonlinear- pseudo-1st-order						
	q_e1_ (mg/g)	K_1_ (min^-1^)	R^2^	χ^2^	MSE	SSE
ARS	97.867	0.047	0.997	0.211	3.43361	20.602

Nonlinear- pseudo-2nd-order						
	q_e2_ (mg/g)	K_2_ (min^-1^)	R^2^	χ^2^	MSE	SSE
ARS	111.357	5.568 × 10^–5^	0.991	7.317	135.839	815.034

Elovich kinetic model						
	$\upalpha$	$\upbeta$	R^2^			
ARS	23.745	0.05	0.939			

IPD						
	K_diff_	R^2^				
ARS	5.015	0.911				

The intraparticle diffusion plot of ARS does not pass through the origin, indicating that IPD is not the sole rate-limiting step and that other factors control the adsorption rate. Showing that the adsorption process occurred in three stages (Fig. S3c). In the first stage, the slope was steep because ARS dye molecules were quickly adsorbed onto the outer surface of the SCB adsorbent, where many active sites remained available. This represented boundary layer diffusion. In the second stage, the slope decreased as the adsorption rate slowed, since the dye molecules began to diffuse into the pores of the SCB adsorbent, a process that takes longer. Finally, the line became almost flat, indicating that equilibrium had been reached and that most active sites were occupied, leading to a significant decrease in the adsorption rate.

##### Effect of temperature and thermodynamic studies of ARS adsorption by SCB

To evaluate the effect of temperature on the adsorption of ARS onto SCB, experiments were conducted over 25–45 °C using 0.005 g of SCB at pH 2, with an initial dye concentration of 50 mg/L and a contact time of 120 min. The results revealed that adsorption capacity decreased with increasing temperature, indicating that the process is exothermic.

Thermodynamic parameters were obtained from the plot of ln Kc versus 1/T, as illustrated in Fig. S4, and are summarized in Table 5. The negative value of ΔH°_ads_ (− 90.248 kJ/mol) confirms the exothermic nature of the adsorption process, and its magnitude falls within the typical chemisorption enthalpy range (40–120 kJ/mol). However, considering the involvement of electrostatic attraction, hydrogen bonding, π–π interactions, and pore-related effects, the adsorption processes are considered physicochemical, involving both chemical and physical interactions. The negative values of ΔG°_ads_ (− 13.256, − 10.513, and − 8.301 kJ/mol) indicate that ARS adsorption onto SCB is spontaneous across the temperatures examined. Furthermore, the negative value of ΔS°_ads_ (− 254.447 J/mol K) suggests a reduction in randomness at the solid–liquid interface during adsorption.

**Table 5:** Thermodynamic parameters for the adsorption of ARS onto SCB biosorbent.

	T (K)	K_C_	ΔG°_ads_ (KJ/mol)	ΔH°_ads_ (KJ/mol)	ΔS°_ads_ (J/mol K)
SCB@ARS	303	192.932	-13.256	- 90.248	- 254.447
	313	56.824	-10.513		
	323	21.998	-8.301		

#### Sequential removal of CV and MG dyes using SCB@ARS

##### Point of zero charge (PZC) of SCB@ARS

**Fig. 11:**
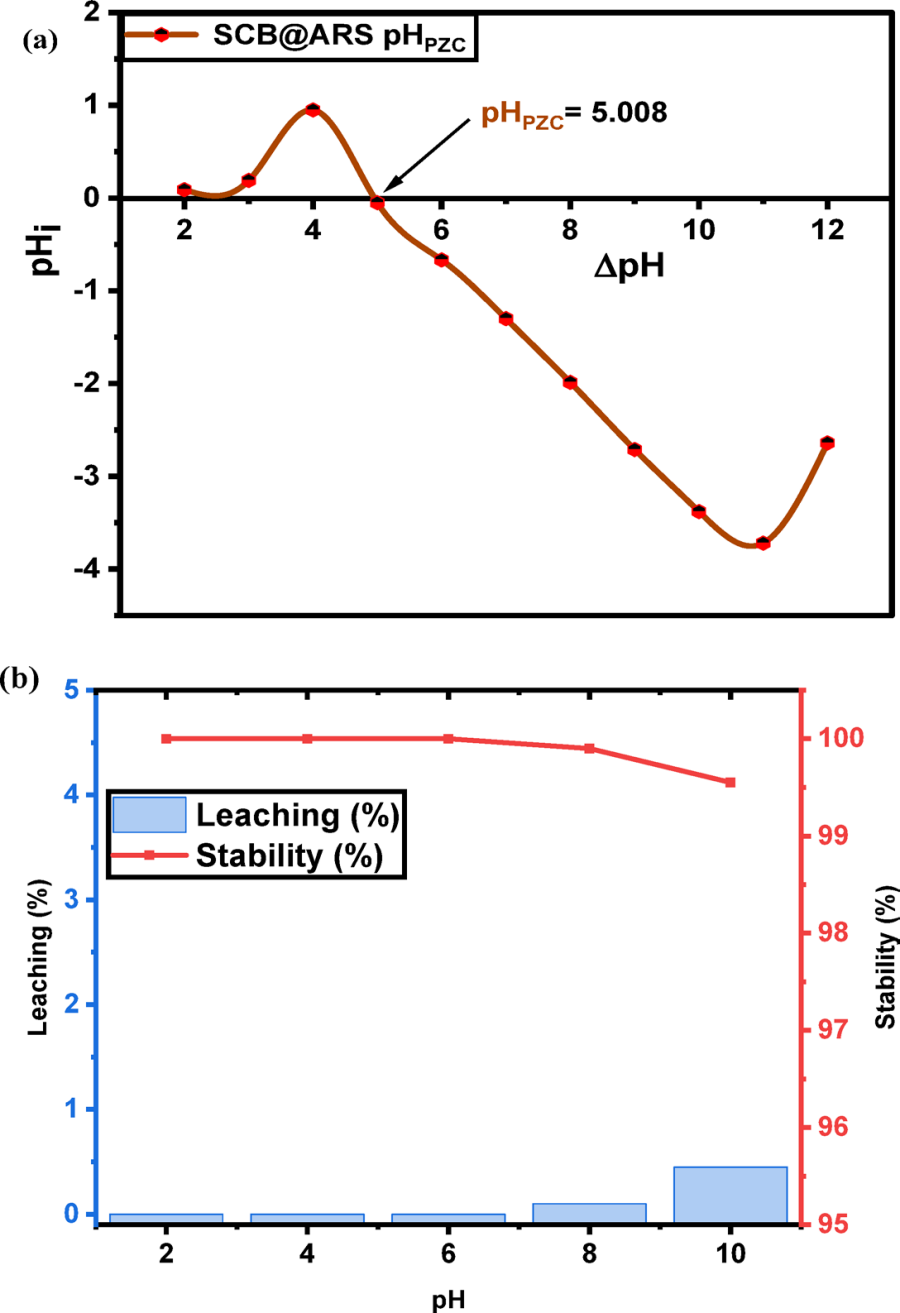
**(a)** The pH_pzc_ value of SCB@ARS adsorbent and **(b)** leaching stability of ARS from SCB@ARS.

As shown in Fig. 11, the pH_pzc_ value for SCB@ARS biosorbent was 5.008. Therefore, when the pH is above 5.008, the SCB@ARS surface is negatively charged, favoring the adsorption of the cationic dyes, CV, and MG.

##### Leaching investigation

As shown in Fig. 11b, no detectable leaching was observed across all tested pH levels, indicating high stability of the immobilized ARS particles in SCB.

##### Effect of pH

**Fig. 12:**
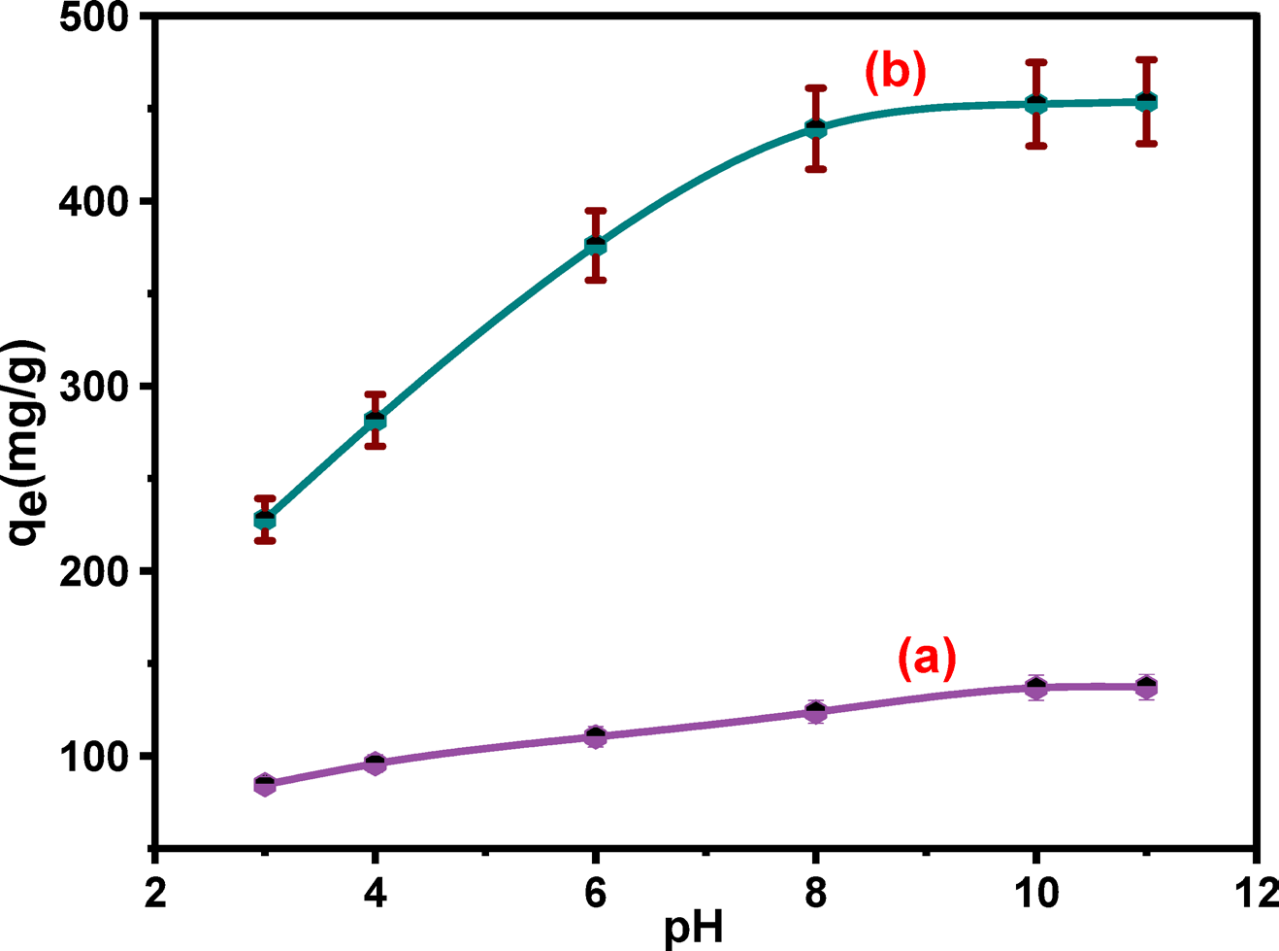
Effect of pH of **(a)** CV and **(b)** MG adsorption onto SCB@ARS biosorbent (conditions: 10 mL aqueous solution of 150 mg/L of CV and 250 mg/L of MG and 0.005 g of SCB@ARS for 120 min).

The impact of solution pH on the removal of the cationic dyes CV and MG using SCB@ARS was investigated over the range 3–11, as shown in Fig. 12.

The investigation was carried out using 0.005 g of SCB@ARS in 10 mL of 250 mg/L MG and 0.01 g of SCB@ARS in 10 mL of 150 mg/L CV, both at room temperature, with shaking for 120 min.

The adsorption capacity rose from 84.559 to 136.841 mg/g for CV and from 227.76 to 452.258 mg/g for MG as pH increased from 3 to 10. Beyond pH 10, these capacities stayed almost the same. This is due to the SCB@ARS surface charge: above pH_pzc_ (5.008), the surface turns negative and attracts cationic dyes. Therefore, pH 10 gives the highest adsorption capacity for CV and MG.

##### Effect of the dose of SCB@ARS biosorbent

To examine how the amount of SCB@ARS biosorbent affects the adsorption process, dosages ranging from 0.0025 to 0.02 g were applied to 10 mL of dye solutions containing 150 mg/L CV and 250 mg/L MG, followed by shaking for 120 min at pH 10 and room temperature.

**Fig. 13:**
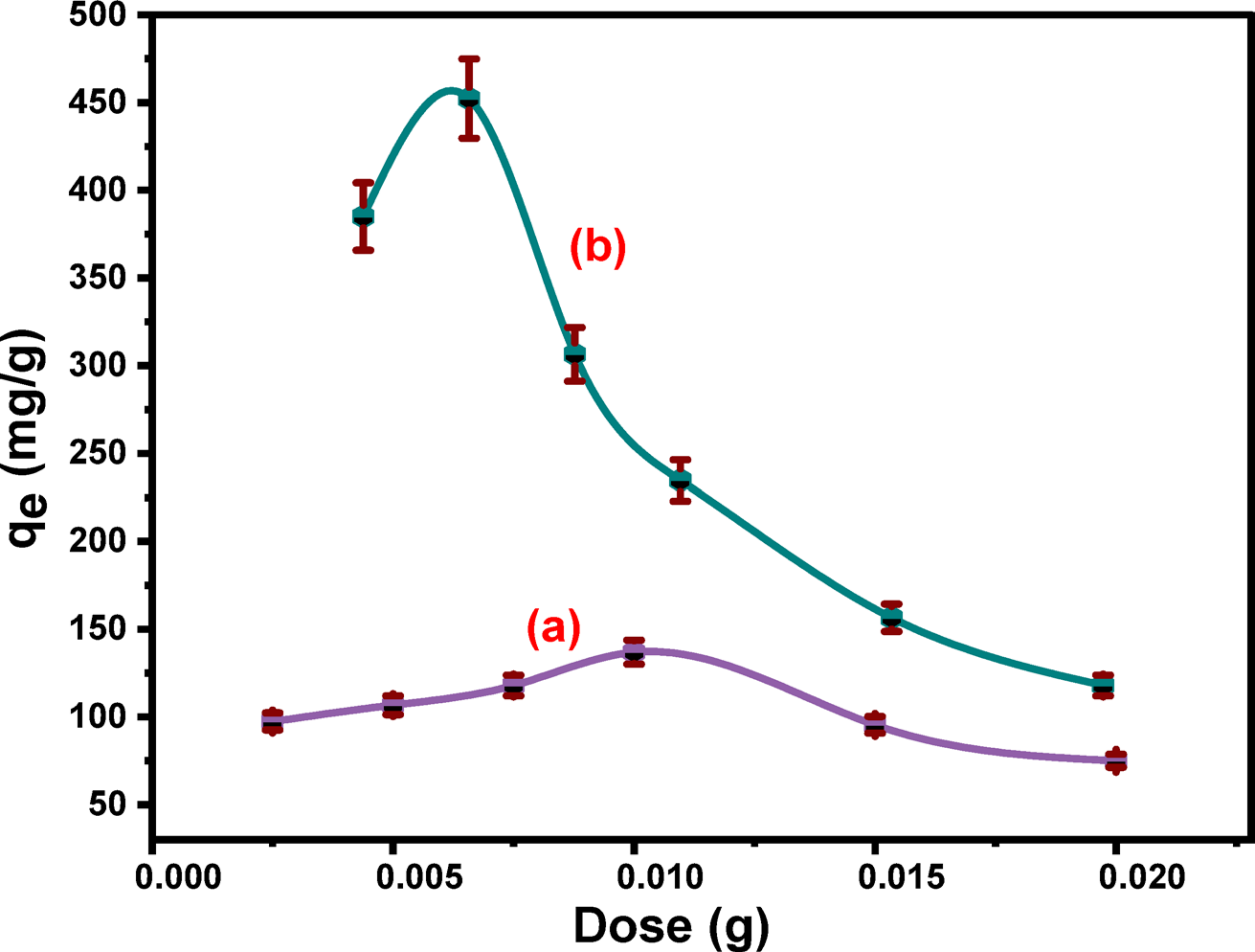
Effect of dosage of **(a)** CV and **(b)** MG adsorption by SCB@ARS biosorbent (conditions: 10 mL aqueous solution of 150 mg/L of CV and 250 mg/L of MG for 120 min at pH 10).

The data presented in Fig. 13 indicated that the adsorption capacity of CV increased with increasing SCB@ARS dosage, reaching its maximum value (136.919 mg/g) at 0.01 g compared to 97.38 mg/g at 0.0025 g. Meanwhile, MG achieved its highest capacity (452.258 mg/g) at a lower dosage of 0.005 g, up from 385 mg/g at 0.0025 g with the SCB@ARS adsorbent. Beyond these dosages, a noticeable decline in adsorption capacity was observed for both dyes.

##### Effect of initial dye concentration and isotherm studies

The effect of initial dye concentration was studied between 50–300 mg/L by preparing 10 mL solutions of CV and MG at pH 10. SCB@ARS dosages of 0.01 g for CV and 0.005 g for MG were used; then, the mixtures were shaken for 120 min at room temperature for both dyes.

**Fig. 14:**
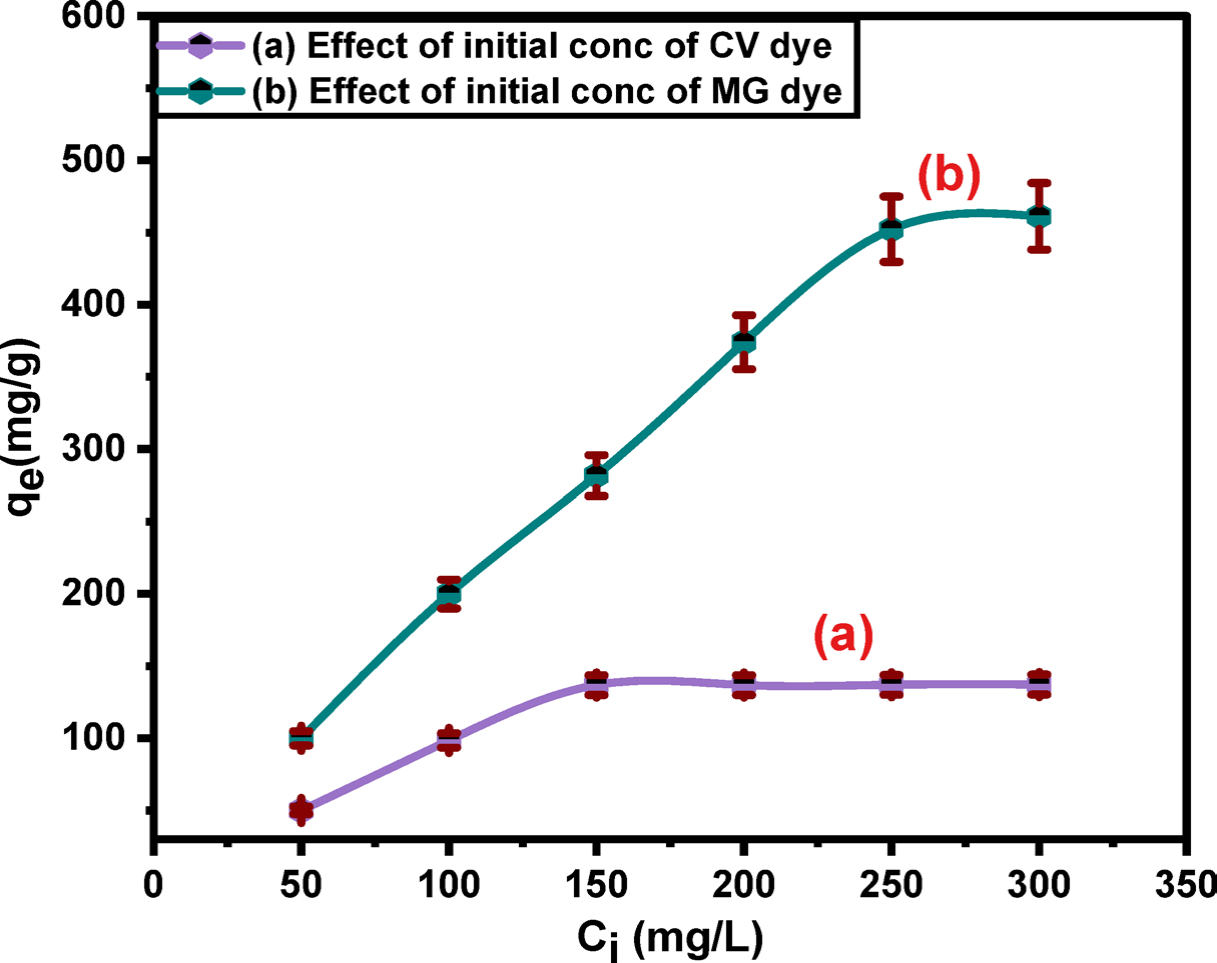
Effect of initial concentration of **(a)** CV, and **(b)** MG adsorption by SCB@ARS biosorbent (conditions: 0.005 g of SCB@ARS and shaking time of 120 min at pH 10).

As shown in Fig. 14, the adsorption capacity increased from 50 to 136.841 mg/g for CV at 50–150 mg/L, and from 99.99 to 452.258 mg/g for MG at 50–250 mg/L. At higher concentrations, the adsorption capacity remained nearly constant, indicating that equilibrium was reached at 150 mg/L for CV and 250 mg/L for MG.

The isotherm models, including the nonlinear and linear Freundlich, the nonlinear and linear Langmuir, and the Dubinin–Radushkevich (D–R), were also applied to the adsorption of CV and MG dyes by SCB@ARS. Fig. S5(a–h) presents the plots for each model, and the corresponding parameter values (q_m_, K_L1_, K_L2,_ R_L_, n1, K_F1_, n2, K_F2,_ and k_DR_) are listed in Table 6.

**Table 6:** Langmuir, Freundlich, and D–R isotherm constants, correlation coefficients, and error functions of SCB@ARS biosorbent for CV and MG dyes.

Linear Langmuir isotherm constants							
	K_L_(L/g)	q_m_(mg/g)	R^2^	R_L_	χ^2^	MSE	SSE
CV	4.282	137.363	0.999	1.554 × 10^–3^	0.0119	0.272	1.635
MG	0.481	473.934	0.992	8.247 × 10^–3^	5.948	469.848	2819.094

Linear Freundlich isotherm constants							
	K_F_	n	R^2^	χ^2^	MSE	SSE	
CV	88.015	9.3826	0.921	7.346	184.145	1104.869	
MG	253.978	7.0028	0.888	54.712	4752.586	28,515.514	

D–R isotherm constants							
	K	E (kJ/mol)	q_m_ (mg/g)	R^2^			
CV	7.249 × 10^–9^	8.305	128.590	0.90211			
MG	7.729 × 10^–9^	8.043	385.291	0.90961			

Nonlinear Langmuir isotherm constants							
	K_L_(L/g)	q_m_(mg/g)	R^2^				
CV	217.44	129.4	0.8193				
MG	39.49	391.79	0.732				

Nonlinear Freundlich isotherm constants							
	K_F_	n	R^2^				
CV	95.4	11.96	0.919				
MG	257.3	7.14	0.908				

The linear Langmuir model, with higher correlation coefficients (R^2^ = 0.999 for CV and 0.992 for MG) and lower errors than other models, best fits the equilibrium data, suggesting monolayer adsorption of CV and MG on a homogeneous SCB@ARS surface. R_L_ values (1.554 × 10^–3^ for CV and 8.247 × 10^–3^ for MG) confirmed a favorable process, and the D–R mean free energy (E_DR_ = 8.305 kJ/mol for CV and 8.247 kJ/mol for MG) indicated physicochemical nature, including both physical and chemical interactions.

##### Effect of contact time and kinetic studies

**Fig. 15:**
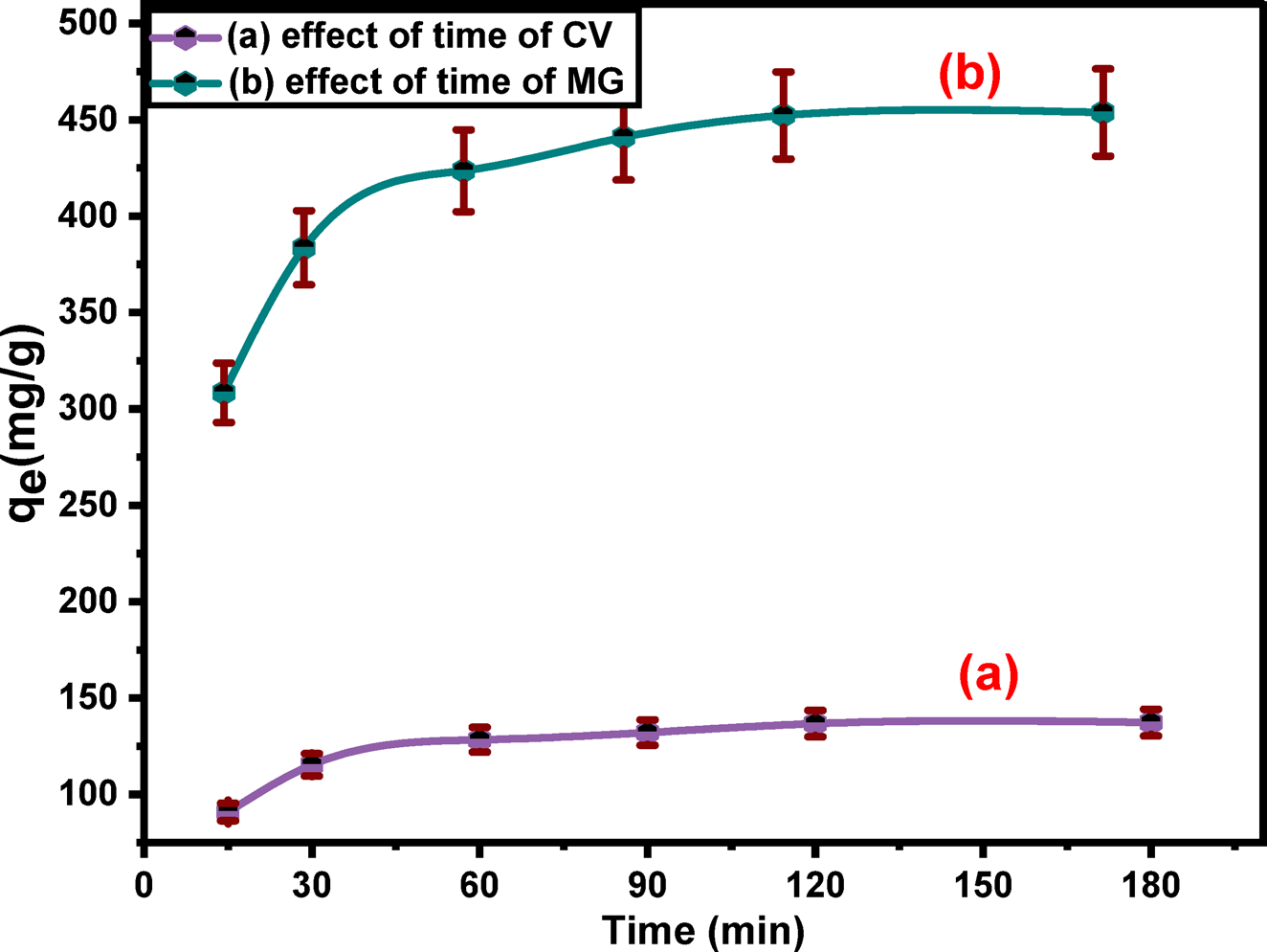
Effect of time on **(a)** CV and **(b)** MG adsorption by SCB@ARS adsorbent (conditions: 10 mL aqueous solution of 150 mg/L of CV and 250 mg/L of MG and 0.005 g of SCB@ARS at pH 10).

The effect of contact time on the adsorption of CV and MG onto the SCB@ARS surface was investigated using 10 mL dye solutions (150 mg/L for CV, 250 mg/L for MG) at pH 10, with adsorbent doses of 0.01 g for CV and 0.005 g for MG. As shown in Fig. 15**,** the adsorption capacity (q_e_) rose sharply at first, increasing from 90.909 to 136.841 mg/g for CV and from 308.378 to 452.258 mg/g for MG within 120 min. This rapid rise was due to the high availability of active sites on the SCB@ARS surface. After 120 min, the concentration remained steady, indicating equilibrium had been reached as active sites became saturated. Therefore, 120 min was considered the optimal contact time for the removal of CV and MG by SCB@ARS.

Three kinetic models—pseudo-1^st^-order (linear and nonlinear), pseudo-2^nd^-order (linear and nonlinear), IPD, and Elovich—were used to describe the adsorption process. **Fig. S6(a-j)** presents the results. The kinetic parameters and R^2^ values are in Table 7.

**Table 7:** Kinetic parameters with error functions for the adsorption of CV and MG by SCB@ARS.

Adsorbates	Linear- pseudo-1st-order					
	q_e1_ (mg/g)	K_1_ (min^-1^)	R^2^	χ^2^	MSE	SSE
CV	146.413	8.934	0.992	3.755	91.623	549.739
MG	480.769	8.236	0.996	10.145	812.877	4877.263

Linear- pseudo-2nd-order						
	q_e2_ (mg/g)	K_2_ (min^-1^)	R^2^	χ^2^	MSE	SSE
CV	143.885	8.998 × 10^–3^	0.999	2.069	49.618	297.708
MG	473.934	2.914 × 10^–4^	0.999	5.948	469.849	2819.094

Nonlinear- pseudo-1st-order						
	q_e1_ (mg/g)	K_1_ (min^-1^)	R^2^	χ^2^	MSE	SSE
CV	133.989	0.0717	0.967	0.364	8.128	48.769
MG	443.029	0.07462	0.958	1.1535	85.174	511.047

Nonlinear- pseudo-2nd-order						
	q_e2_ (mg/g)	K_2_ (min^-1^)	R^2^	χ^2^	MSE	SSE
CV	145.608	8.011 × 10^–4^	0.992	3.168	76.878	461.267
MG	479.586	2.598 × 10^–4^	0.995	9.343	746.819	4480.918

Elovich kinetic model						
	$\upalpha$	$\upbeta$	R^2^			
CV	238.36	0.054	0.914			
MG	1060.867	0.017	0.919			

IPD						
	K_diff_	R^2^				
CV	4.897	0.916				
MG	15.244	0.919				

According to the R^2^ values obtained from the investigated kinetic models, the adsorption data showed better agreement with the linear pseudo-2nd-order model (R^2^ = 0.999). However, the nonlinear pseudo-1st-order model, along with the calculated q_e_ values and error functions, showed better agreement. This difference between linear and nonlinear approaches indicates that a single model cannot describe the kinetic behavior and may reflect the complex nature of ARS adsorption in SCB, involving multiple interactions and physicochemical contributions.

The intraparticle diffusion plots of CV and MG don’t pass through the origin, indicating that IPD is not the sole rate-limiting step and that there are other controls on the adsorption rate besides it. The IPD plots showed three linear segments, indicating that the adsorption of CV and MG occurs in multiple steps. In the first stage, the slope increased sharply as dye molecules quickly adsorbed onto the outer surface of the SCB@ARS adsorbent (boundary-layer diffusion). In the second stage, the slope decreased as adsorption slowed; dye molecules then diffused into SCB@ARS pores, a slower process. In the final stage, the line flattened, indicating equilibrium and saturation of active sites.

##### Effect of temperature and thermodynamic studies

To study the effect of temperature on the adsorption of CV and MG, 10 mL of 150 mg/L CV and 250 mg/L MG were placed in two bottles containing 0.01 g of SCB@ARS for CV and 0.005 g for MG at different temperatures between 25 and 45 °C. The solutions were adjusted to pH 10, and the bottles were then shaken in a balanced shaker at 150 rpm for 30 min.

It was noted that the adsorption capacity on SCB@ARS increased with increasing temperature. This trend is illustrated in Fig. S7, which plots the natural logarithm of the equilibrium constant (ln KC) versus the inverse temperature (1/T) in Kelvin.

Furthermore, as shown in Table 8, the positive ΔH°_ads_ values indicate that adsorption for both dyes is endothermic, which explains the observed increase in adsorption capacity with increasing temperature. Since ΔH°_ads_ values (80.687 kJ/mol for CV and 88.794 kJ/mol for MG) fall within the range of strong interactions/chemisorption (40–120 kJ/mol), the adsorption processes are suggested to be chemisorption. However, considering the involvement of electrostatic attraction, hydrogen bonding, π–π interactions, and pore-related effects, the adsorption processes are considered physicochemical, involving both chemical and physical interactions. Additionally, the positive ΔS°_ads_ values indicate increased randomness at the solid/liquid interface during adsorption, while the negative ΔG°_ads_ values confirm the feasibility and spontaneity of the processes.

**Table 8:** Thermodynamic parameters for the adsorption of CV and MG onto SCB@ARS biosorbent.

	T (K)	K_C_	ΔG°_ads_ (KJ/mol)	ΔH°_ads_ (KJ/mol)	ΔS°_ads_ (J/mol K)
CV	303	7.715	-5.147	80.687	283.189
	313	19.877	-7.781		
	323	53.685	-10.699		
MG	303	16.676	-7.089	88.794	316.317
	313	46.857	-10.011		
	323	141.184	-12.881		

##### Effect of ionic strength

**Fig. 16:**
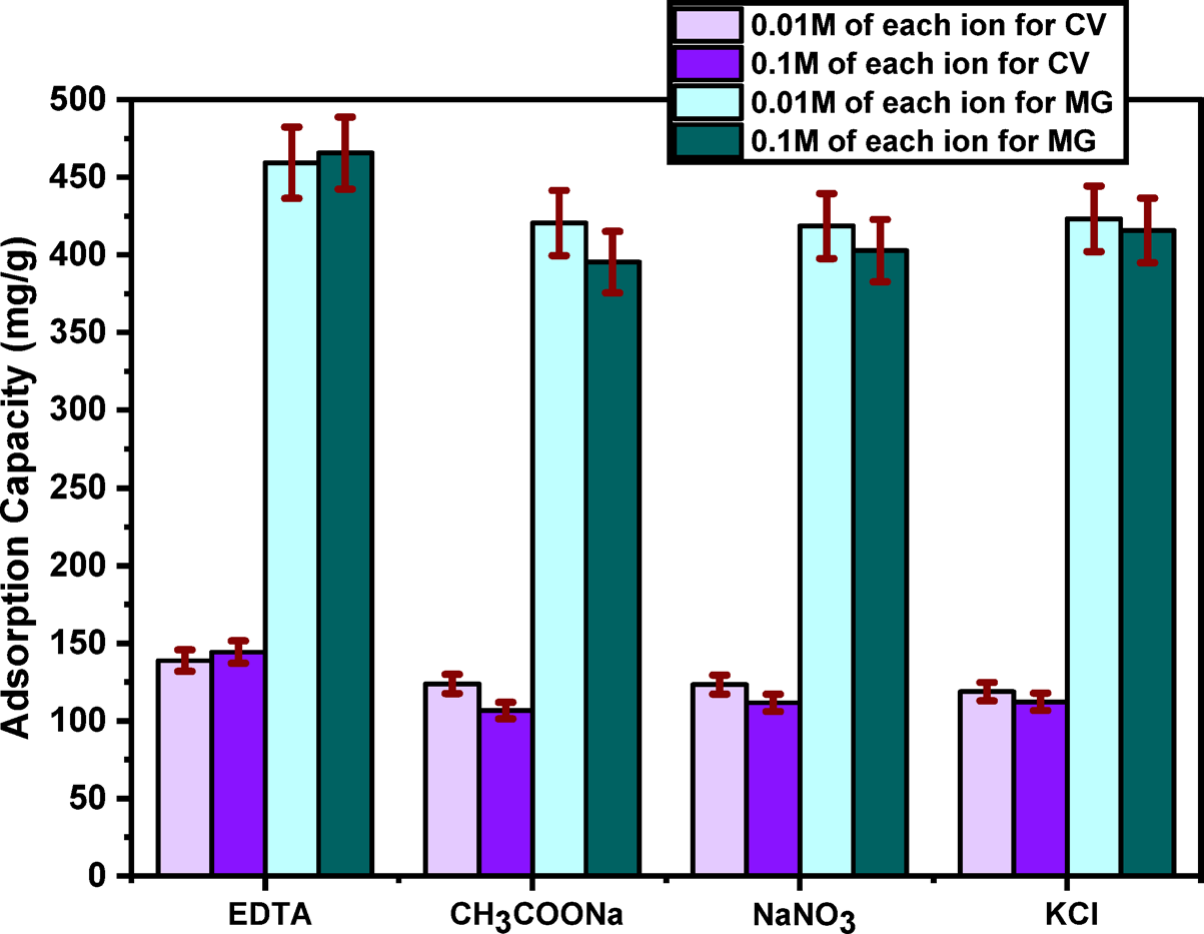
Effect of ionic strength on the CV and MG adsorption ((conditions: 10 mL aqueous solution of 150 mg/L of CV and 250 mg/L of MG and 0.005 g of SCB@ARS for 120 min at pH 10).

Industrial wastewater contains large concentrations of various solutes; therefore, ionic strength is an essential factor to consider. It was tested with a variety of ions, including EDTA, CH3COONa, NaNO_3_, and KCl at 0.01 and 0.1 M, as presented in Fig. 16.

Building on these tests, CV and MG adsorption on SCB@ARS increased slightly with EDTA, because EDTA chelated interfering metal ions, thus preventing them from occupying the active sites on SCB@ARS. As a result, the active sites retained more negatively charged groups that could interact with the dye molecules, thereby improving adsorption performance.

As expected, the adsorption capacities of CV and MG decreased in the presence of salt ions due to competition between Na^+^ and K^+^ cations and the cationic dyes for the negatively charged functional groups on the SCB@ARS surface. Because Na^+^ (≈1.02 Å) and K^+^ (≈1.38 Å) have smaller ionic sizes and higher mobility, they can more readily access and occupy adsorption sites than the bulky triphenylmethane cationic dyes. Consequently, the adsorption capacity decreased further as the salt molarity increased from 0.01 M to 0.1 M^76^.

##### Removal of CV and MG in a multi-contaminant system

**Fig. 17:**
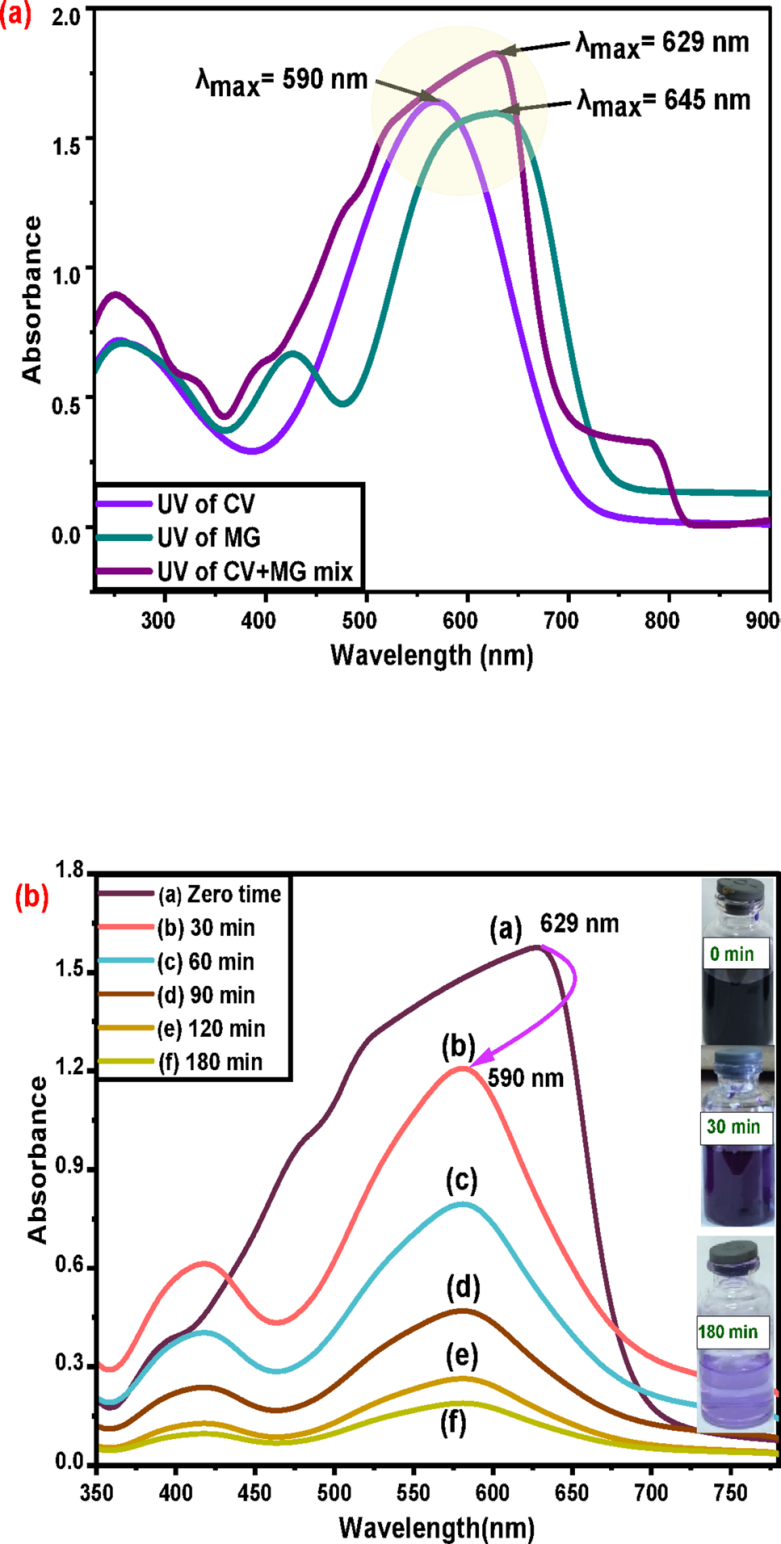
UV data of **(a)** CV-MG mix compared with CV and MG; **(b)** CV-MG mix after adsorption by SCB@ARS at different periods of time (conditions: 0.01 g of SCB@ARS and 10 mL of mixture (150 mg/L CV, 250 mg/L MG) at pH 10).

As the dyes are present in mixed forms in actual polluted water sources, it was essential to apply the SCB@ARS adsorbent for the combined system removal of the investigated dyes. As shown in Fig. 17a, a new λ_max_ appeared at 629 nm for the studied mixture (CV-MG) that was not far away from the λ_max_ of every single dye (590 nm for CV and 645 nm for MG), but in between, which demonstrated the equivalent selectivity toward the removal of the two dyes in the binary system by SCB@ARS. As shown in Fig. 17b, after 30 min, the peak of the mixture shifted to 590 nm with lower intensity, indicating that MG was adsorbed first, followed by CV. Moreover, the absorbance, an indication of the dye’s concentration, decreased markedly with increasing time. The system reached equilibrium within 2 h, with no significant increase in removal capacity thereafter, confirming the effective removal of the dyes by SCB@ARS in the binary system.

##### Application

###### In natural water samples

The application experiments of the SCB@ARS were conducted by adsorbing CV and MG at 50–150 and 50–250 mg/L, respectively, from tap water, Nile water, seawater, and wastewater (before and after treatment) samples to assess the applicability of SCB@ARS in real samples. As shown in Table 9**,** the recoveries ranged from 90.345% to 105.940% with RSD < 1.50. These results demonstrated that SCB@ARS adsorbent could be applied to remove cationic dyes from real water samples. These results, with high recovered concentrations and low RSD (%), demonstrate that SCB@ARS can be effectively used to remove CV and MG from aqueous environments in practice.

**Table 9:** Analytical results of the adsorption of CV and MG in natural water samples employing SCB@ARS as an adsorbent. (n = 3).

Sample	Pollutant	Spiked (µg/mL)	Measured(µg/mL)	Recovered (µg/mL)	Recovery (%)	RSD (%)
Waste water before treatment	CV	0.00	0.00	0.00	0.00	0.00
		5	0.03	4.97	99.4	0.94
		10	0.1	9.9	99	1.34
		50	0.552	49.446	98.896	1.16
		100	3.114	96.886	98.351	0.81
		150	16.265	133.735	97.730	0.60
	MG	0.00	0.00	0.00	0.00	0.00
		5	0.13	4.87	97.4	0.63
		10	0.08	9.92	99.2	1.45
		50	0.541	49.459	98.928	0.94
		100	1.021	98.979	99.011	1.33
		150	5.615	144.385	102.482	0.76
		200	7.105	192.895	103.214	1.02
		250	10.438	239.562	105.940	0.12
Waste water after treatment	CV	0.00	0.00	0.00	0.00	0.00
		5	0.16	4.84	96.8	1.11
		10	0.34	9.66	96.6	0.99
		50	1.031	48.969	97.938	1.21
		100	4.922	95.078	96.516	0.45
		150	18.393	131.607	96.175	1.08
	MG	0.00	0.00	0.00	0.00	0.00
		5	0.05	4.95	99	0.94
		10	0.23	9.77	97.7	0.6
		50	1.043	48.957	97.924	0.57
		100	1.551	98.449	98.481	1.28
		150	9.715	140.285	99.572	0.09
		200	11.835	188.165	100.637	0.16
		250	15.934	234.066	103.509	1.18
Sea water	CV	0.00	0.00	0.00	0.00	0.00
		5	0.09	4.91	98.2	0.13
		10	0.18	9.82	98.2	0.54
		50	1.832	48.168	96.336	1.03
		100	6.781	93.219	94.644	0.22
		150	22.131	127.869	93.443	0.99
	MG	0.00	0.00	0.00	0.00	0.00
		5	0.15	4.85	97	0.98
		10	0.46	9.54	95.4	0.32
		50	2.502	47.498	95.006	1.09
		100	3.913	96.087	96.118	0.10
		150	12.427	137.573	97.647	0.57
		200	16.398	183.602	98.197	1.11
		250	26.106	223.894	99.012	0.00
Tap water	CV	0.00	0.00	0.00	0.00	0.34
		5	0.21	4.79	95.8	0.43
		10	0.53	9.47	95.7	1.02
		50	2.114	47.886	95.772	1.38
		100	8.345	91.655	93.941	0.84
		150	25.057	124.943	91.305	0.05
	MG	0.00	0.00	0.00	0.00	0.00
		5	0.29	4.71	94.2	1.13
		10	0.86	9.14	91.4	0.26
		50	4.832	45.168	90.345	0.42
		100	9.147	90.853	90.882	0.02
		150	20.981	129.019	91.576	1.41
		200	26.654	173.346	92.711	0.39
		250	37.960	212.04	93.769	0.17
Gentian violet	CV	50	0.9	49.1	98.2	0.91
		100	2.8	97.2	98.67	0.32
		150	14.25	135.75	99.204	

###### In pharmaceutical samples

As presented in Table 9, the SCB@ARS efficiency toward CV and MG recovery from pharmaceutical samples was evaluated using Gentian violet. SCB@ARS exhibited high adsorption efficiency for Gentian Violet, achieving 90.501% removal with a recovery rate of 99.204%. These findings indicate that the SCB@ARS is a promising and efficient adsorbent for the removal of cationic dyes from various sample types.

#### Proposed sequential adsorption mechanism

**Fig. 18:**
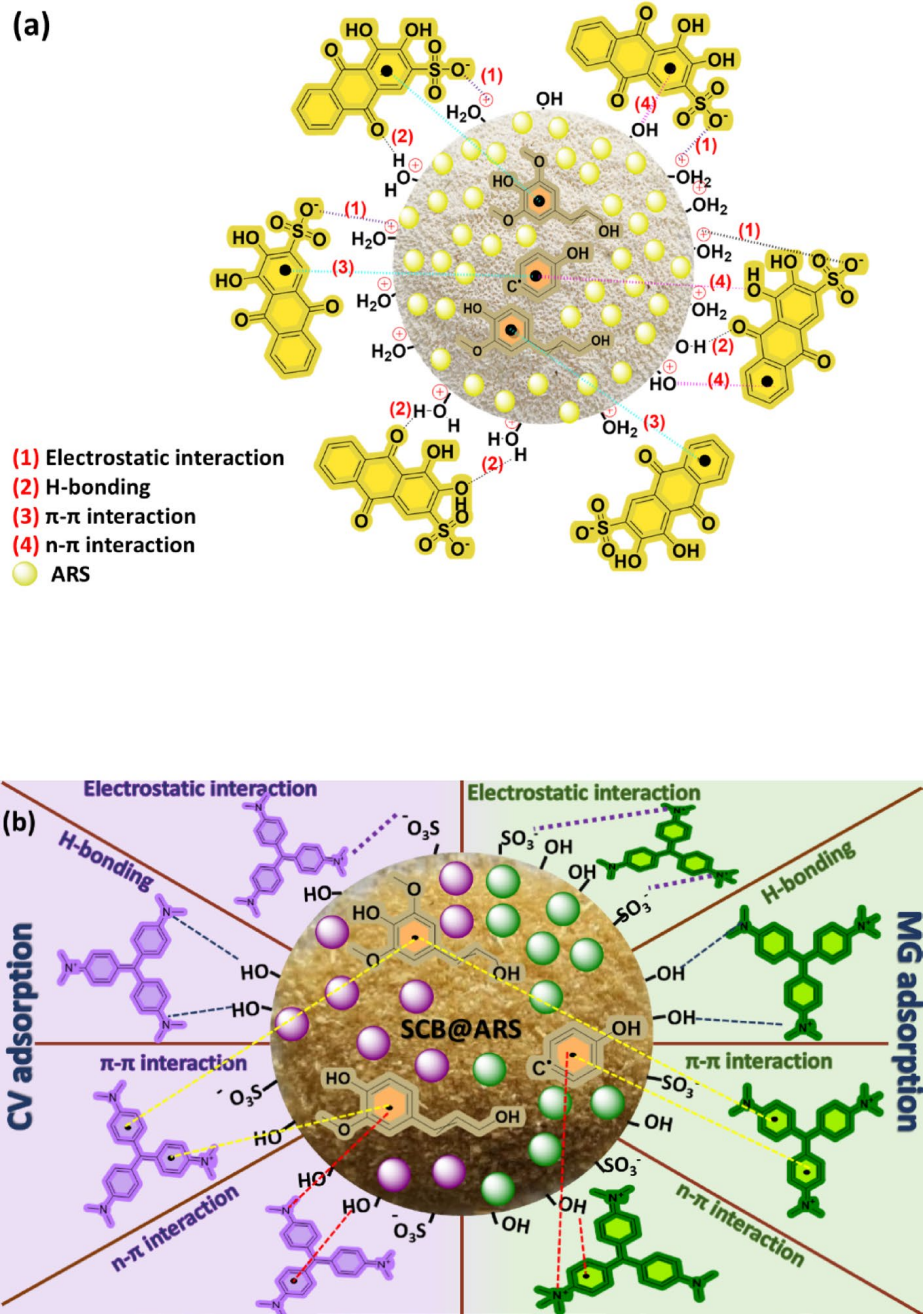
Proposed adsorption mechanism of **(a)** ARS onto SCB and **(b)** CV and MG onto SCB@ARS.

This study examines the sequential adsorption of cationic CV and MG after the adsorption of the anionic ARS dye. Multiple factors influence dye adsorption on SCB and SCB@ARS surfaces^77^, including pore filling, electrostatic attraction, n–π interactions, hydrogen bonding, and π–π interactions. Additional hydrophobic forces may also contribute. Previous studies have identified electrostatic interactions as the primary mechanism. To investigate the possible mechanism of the current sequential adsorption, EDX (Fig. 3), SEM (Fig. 5), surface charge (Fig. 7 and Fig. 11b), and FTIR (Fig. 4) were evaluated. The adsorption mechanism of ARS onto SCB and CV/MG onto SCB@ARS was elucidated in light of the effective groups available on SCB and SCB@ARS, as presented in Fig. 18. SCB is composed of cellulose, hemicellulose, and lignin, which confer abundant OH groups enabling the adsorption of anionic species. Besides these groups, the SCB@ARS has added a functional group (SO_3_^-^) after the ARS, which enhances adsorption efficiency toward cationic dyes (CV and MG). The proposed binding mechanism, highlighting interactions between ARS molecules and O− functionalities on SCB, as well as between MG and CV molecules and O− functionalities on SCB@ARS in several ways, such as:

- The pH of the CV, MG, and ARS solutions plays a major role in the adsorption process. At pH lower than the SCB’s pH_PZC_, the surface becomes positively charged and electrostatic attraction between the positively charged (OH_2_^+^) groups in acidic medium and the negatively ARS SO_3_^-^ groups. While at pH higher than SCB@ARS’ pH_PZC_, the adsorbent surface becomes negatively charged and the electrostatic attraction between the negative charges of the SCB@ARS (SO_3_^-^) and cationic dyes’ positive charges (NH_2_^+^)^78,79^.
- H-bonding as one bond forms between the –OH groups on the adsorbents’ surface and the O- and N-atoms in ARS, MG, and CV dyes.
- π–π interaction as one bond forms between the electrons in the aromatic ring of the SCB’ lignin units and the π-electrons of the aromatic ring in dye molecules.
- n–π interaction as one bond of the O–atom (–OH) and the π-electrons of the benzene rings.

#### Statistical analysis

**Fig. 19:**
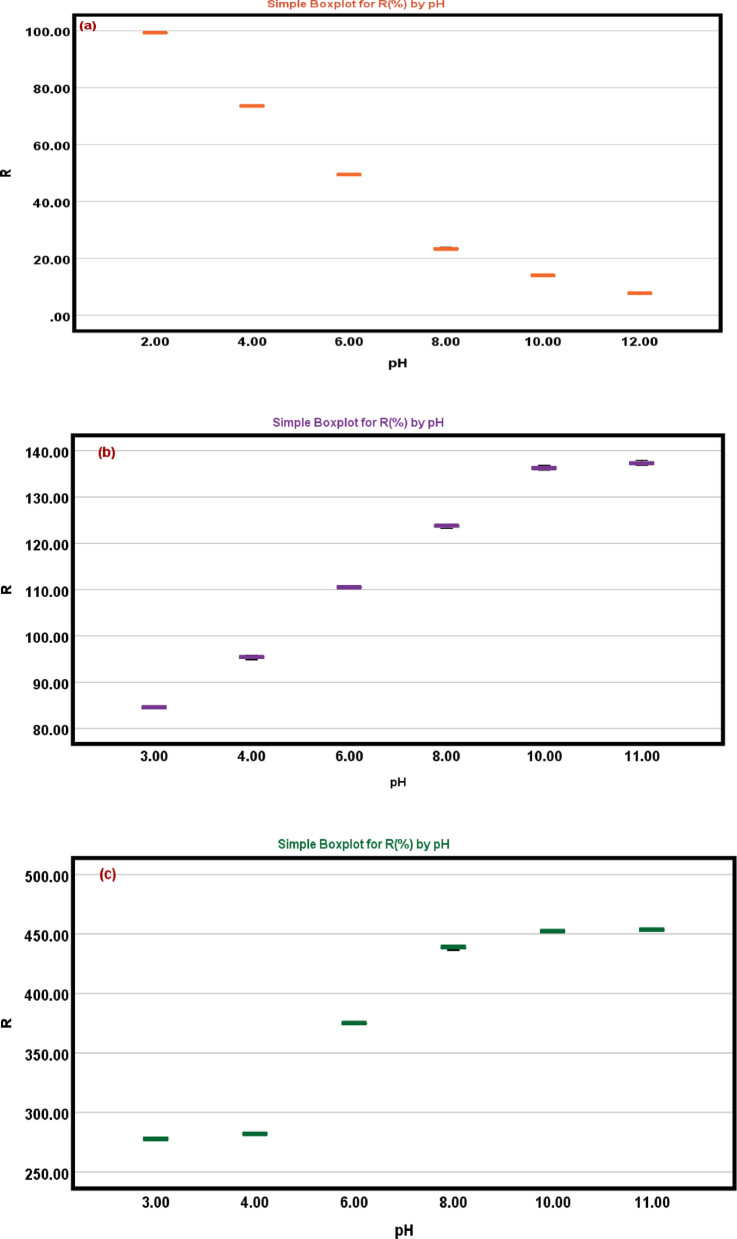
Simple boxplot of R(%) by pH for **(a)** ARS adsorption using SCB, **(b)** CV adsorption using SCB@ARS, and **(c)** MG adsorption using SCB@ARS.

pH is an important parameter for controlling the adsorption of the ARS using SCB and (CV/MG) using SCB@ARS. To uncover how pH truly influences ARS adsorption using SCB, along with CV and MG using SCB@ARS, a one-way analysis of variance (ANOVA) was conducted using IBM SPSS Statistics for Windows, Version 25.0 (IBM Corp., Armonk, NY, USA). Available at: (https://www.ibm.com/legal/copyright-trademark). This analysis determines whether variations in the pH yield statistically significant effects. Boxplots for pH are presented in Fig. 19** (a-c)**, respectively. In the pH boxplot for ARS (Fig. 19a), the R(%) was observed at a pH value of 2, followed by a decrease at higher pH values. In the pH boxplot for CV and MG (Fig. 19b), the R(%) toward them increased as the pH rose from 2 to 10, then remained constant. The ANOVA results for the three dyes, shown in Table 10**,** indicate that the pH parameter significantly affects ARS adsorption by SCB and (CV and MG) by SCB@ARS, as evidenced by high F-values and P-values below 0.001. These findings confirm that the changes in R(%) with respect to pH are statistically significant, establishing this variable as a critical parameter for ARS adsorption by SCB and (CV and MG) by SCB@ARS.

**Table 10:** ANOVA statistical analysis for pH and dose parameters (n = 3).

ANOVA					
ARS adsorption using SCB					
	Sum of squares	Degree of freedom	Mean square	F	P
Between groups	19,838.393	5	3967.679	56,006.56	0.000
Within groups	0.850	12	0.071		
Total	19,839.243	17			

CV adsorption using SCB@ARS					
	Sum of squares	Degree of freedom	Mean square	F	P
Between groups	7089.709	5	1417.942	7991.408	0.000
Within groups	2.129	12	0.177		
Total	7091.838	17			

MG adsorption using SCB@ARS					
	Sum of squares	Degree of freedom	Mean square	F	P
Between groups	102,188.773	5	20,437.755	23,466.65	0.000
Within groups	10.451	12	0.871		
Total	102,199.22	17			

#### Performance of pristine SCB and SCB@ARS adsorbents

To assess the value of the SCB and SCB@ARS adsorbents, a comparison of their maximum adsorption capacity (q_max_) with other reported adsorbents for ARS and (CV and MG) removal, respectively, was obtained, as presented in Table 11**.** It was observed that ARS adsorption by the SCB is reasonably positioned with respect to other mentioned investigations, with a q_max_ of 99.38 mg/g at 25 °C, as it presents a q_max_ value higher than that of the other reported materials’ q_max_. Moreover, the SCB@ARS exhibited excellent adsorption performance for CV and MG, achieving high capacities of 136.919 mg/g and 452.258 mg/g, respectively. It stands out among the adsorbents reported in Table 11 due to its superior uptake, reflecting its abundance of binding sites and strong interactions with the CV and MG dyes, making it highly effective for wastewater treatment. It can be concluded that SCB and SCB@ARS are efficient adsorbents for the adsorption of organic dyes.

**Table 11:** Comparison of the adsorption capacity of CV and MG onto SCB@ARS with previously reported studies.

Adsorbate	Adsorbent	Adsorbent dose	Initial concentration (mg/L)	Equilibrium time (min)	Adsorption capacity (mg/g)	References
ARS	SCB	0.005 g	50	120	99.38	Present study
	Sulfuric acid-modified avocado seeds	3 g/L	50	30	67.08	^80^
	Spirulina platensis algae	1.5 g/L	100	42.5	17.15	^81^
	Zeolite L	0.025 g/L	20–40	120	95.4	^82^
	Flax fiber-based semicarbazide	3 g/L	100	100	18.84	^83^
CV	SCB@ARS	0.01 g	150	120	136.841	Present study
	cassava peels powder (RCP)	1 g	100	60	26.63	^84^
	Peanut husk (PH)	0.1 g	100	10	20.95	^85^
	Trifolium repens stem powder	1 g	70	140	1.952	^86^
	Coconut husk	0.6 g	50	60	0.7283	^87^
MG	SCB@ARS	0.005 g	250	120	452.258	Present study
	Polyacrylonitrile modified by Fe_2_O_3_NPs	0.01 g	30	75	117.803	^88^
	MnFe_2_O_4_/clay composite	0.2 g	125	40	44	^89^
	Silicon powder	0.8 g	25	40	38.639	^90^
	Loofah fibers	0.4 g/L	5	30	18.16	^91^
	Fava bean peel	0.1 g/50 mL	20	30	11.36	^92^

The performance of SCB in the sequential adsorption approach is evaluated against previously published studies to determine the efficiency of the current strategy. As shown in Table 12, the present investigation demonstrates higher adsorption capacities, particularly for the second pollutant, compared to the earlier studies. Moreover, it was observed that the previously reported studies focused on the removal of different natures: one organic and the other inorganic. However, the current investigation concerned the removal of two organic pollutants with different charges.

**Table 12:** Comparison of SCB and SCB@ARS sorption capacity with previously reported sequential adsorption studies.

Adsorbent	Pollutants removed		Adsorbent dosage	Initial concentration (mg/L)	Equilibrium time (min)	Adsorption capacity (mg/g)	Reference
Biochar from Gracilaria Rhodophyta red weeds	1st pollutant	Silver (Ag)	1.0 g/100 mL	50	45	4.7	^93^
	2nd pollutant	Brilliant Green (BG) dye	0.5 g/100 mL	100	60	17.8	
Sheep wool	1st pollutant	Alizarin red S (ARS)	8.0 g/L	100	90	11.65	^94^
	2nd pollutant	Cr(VI)	8.0 g/L	100	120	9.725	
Nano sulfonated poly (glycidyl meth- acrylate) particles (SPGMA)	1st pollutant	CV	10 mg	200	90	174.6	^49^
	2nd pollutant	Cr(VI)	10 mg	100	90	84.6	
		Mn(VII)	10 mg	20	90	11.66	
Sugarcane bagasse	1st pollutant	Alizarin red S (ARS)	0.5 g/l	50	120	99.38	Current study
	2nd pollutant	CV	1 g/L	150	120	136.84	
		MG	0.5 g/L	250	120	452.26	

#### Environmental impact

Using natural materials, such as SCB, as adsorbents for water contaminant removal typically results in a more favorable environmental impact than conventional chemical methods^23,95^. SCB is an environmentally advantageous adsorbent because it is an agricultural waste product. Using agricultural waste not only mitigates disposal challenges but also promotes sustainable approaches to wastewater color removal. The cost-effectiveness of SCB positions it as a practical alternative to traditional adsorbents or chemical treatments, particularly in developing countries where financial limitations are pronounced. Its widespread availability further enhances its applicability across various geographic regions. SCB provides an effective solution for dye remediation in wastewater treatment. Additionally, self-functionalization after ARS adsorption yields SCB@ARS, which has been used as an adsorbent for the removal of cationic CV and MG dyes.

### Limitations

Water treatment processes are often subject to several limitations, including removal capacity, selectivity, stability of the applied material in various media, reusability, economic viability, and environmental challenges. The reusability of the applied material specifically limits the present study.

### Future recommendations

Greater emphasis should be placed on sequential adsorption for the removal of multiple contaminants with different charges, as this approach utilizes low-cost, self-functionalized materials that do not require further modification to enhance adsorption efficiency. Additionally, investigating the removal of various pollutants across multiple systems more accurately simulates real-world water treatment scenarios. Kinetic and isotherm models should be evaluated using error function analysis. Furthermore, the use of eco-friendly, practical, low-cost, and readily available biowastes warrants further exploration. Finally, the application of these methods to actual waste samples should be prioritized.

## Conclusion

**Fig. 20:**
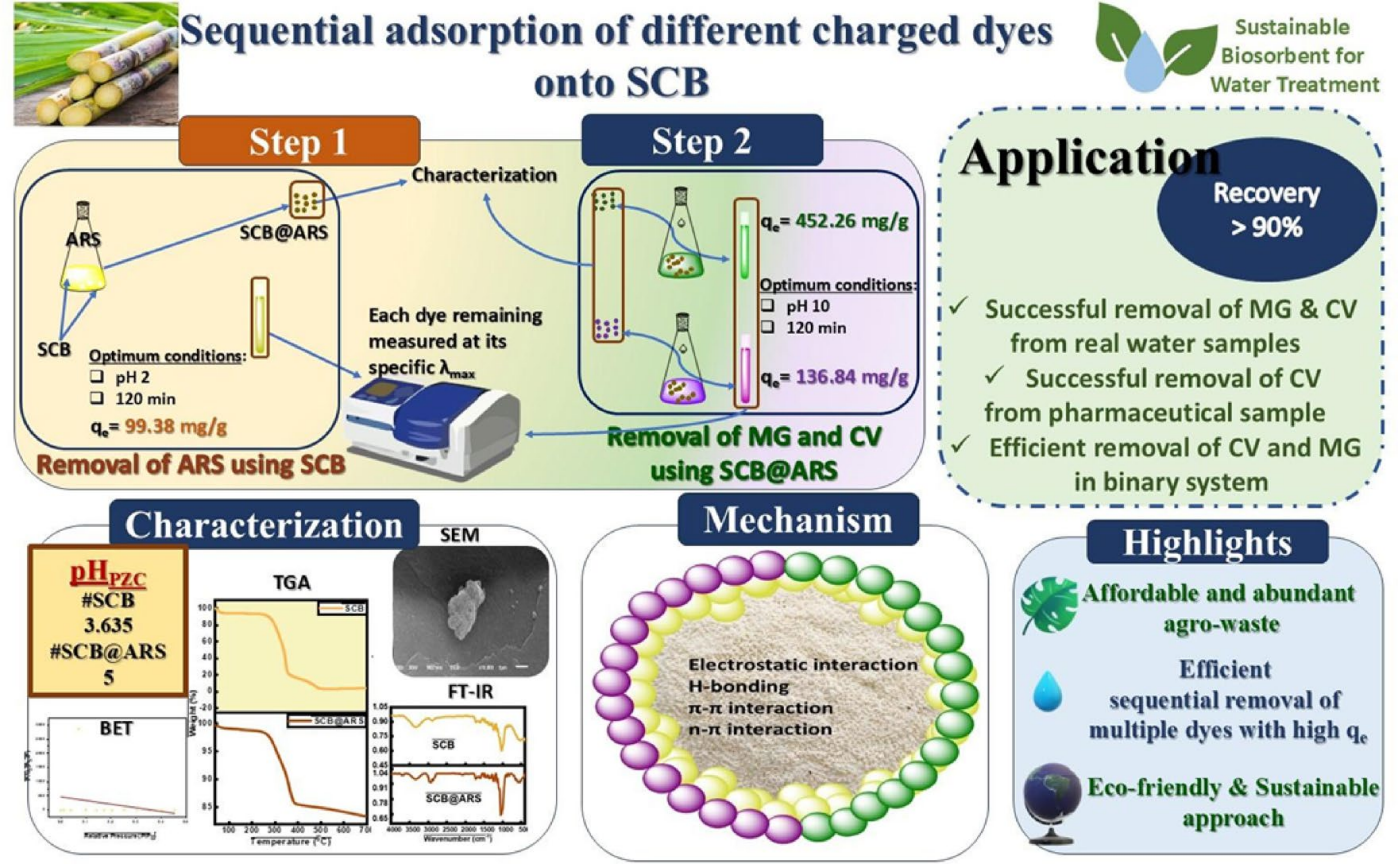
The graphical representation of the sequential adsorption steps, characterization, application, and adsorption mechanism of ARS into SCB and (CV/MG) into SCB@ARS.

This study demonstrates an efficient sequential adsorption process for anionic dyes (ARS) followed by cationic dyes (CV and MG), using cost-effective, abundant agro-waste (SCB). The SCB and SCB@ARS were characterized using physical, chemical, and morphological techniques. SCB achieved effective ARS adsorption, with a q_e_ of 99.38 mg/g at pH 2. SCB@ARS showed high adsorption capacities for CV and MG, with q_e_ values of 136.84 and 117.8 mg/g at pH 10, respectively. The adsorption of all three dyes, as indicated by the kinetic and isotherm, was best described by the linear pseudo-2nd-order and nonlinear pseudo-1st-order kinetic models and the Langmuir isotherm model. Thermodynamic analysis shows that the sequential adsorption process was spontaneous and exothermic. SCB@ARS has been effectively used to remediate both CV and MG from contaminated water sources and CV from environmental and pharmaceutical samples, with recovery (%) exceeding 90%. Moreover, the SCB@ARS was successfully applied to remove CV and MG in a binary system. The adsorption processes were proposed to be mainly due to electrostatic interactions, π-π and n-π interactions, and hydrogen bonding. Although these results are promising, the experiments were performed in a laboratory environment, which may not fully represent the complexity of real wastewater matrices. Figure 20 presents the sequential adsorption steps, characterization, application, and adsorption mechanism of ARS into SCB and (CV/MG) into SCB@ARS.

## Supplementary Information

Below is the link to the electronic supplementary material.

Supplementary Material 1

## Data Availability

All data supporting the findings of this study are available within the paper and its Supplementary Information.
