# Supplementary material for: Sequential removal of oppositely charged multi pollutants from wastewater using sugarcane bagasse
Source: Sci Rep. 2026 Jul 22;16:22945. doi: 10.1038/s41598-026-62305-9 (PMC13392420; doi:10.1038/s41598-026-62305-9)
Supplement: Supplementary file 1 — Supplementary Material 1 [file 41598_2026_62305_MOESM1_ESM.docx]

# **Sequential Removal of Oppositely Charged Multi pollutants from Wastewater Using Sugarcane Bagasse**

**Magda A Akl^1^*, Azza A Fahim^1^ and Aya G Mostafa^1^**

**^1^**Department of Chemistry, Faculty of Science, Mansoura University, Mansoura 31556, Egypt

* To whom correspondence should be addressed: Prof Magda Akl.

email magdaakl@yahoo.com

**S1. Isotherm Investigation**

Three isotherm models, namely the Langmuir, Freundlich, and Dubinin–Radushkevich models, were studied for the adsorption of ARS by SCB and CV, and of MG by SCB@ARS. The Langmuir isotherm postulates that once an adsorbate occupies a site, no further adsorption occurs there, creating a discriminating plateau in the curve, and there is no side commerce or steric interference between adsorbed molecules [1]. The linear model equation and the Langmuir separation factor (R_L_), an important parameter used to calculate affinities between adsorbents and sorbates, are represented by **Eqs. (1)** and **(2)**.

On the other hand, the Freundlich isotherm could be considered an empirical model that involves interactions among adsorbed molecules (multilayer adsorption) on heterogeneous surfaces with a uniform energy distribution. Also, this model showed that the adsorbate concentration on the adsorbent surface increases with increasing solution concentration, without attaining saturation [2]. The linear Freundlich isotherm model is represented by **Eq. (3)**. The Langmuir and Freundlich isotherms, in their nonlinear forms, are represented by **Eq. (4)** and **Eq. (5),** respectively.

The D-R isotherm model demonstrates that adsorption is linked to surface porosity and pore volume, with a focus on the energetic aspects of adsorption. The average free energy of adsorption (E_DR_) derived from the D-R model indicates whether the adsorption mechanism is chemical or physical. 8 < E_DR_ < 16 kJ/mol suggests that the adsorption possesses a chemical nature. If E_DR_ < 8 kJ/mol, the adsorption is physical. The model equation is represented by **Eq. (6)**.

$\frac{C_{e}}{q_{e}}= \frac{1}{K_{L1}q_{m}}+\frac{C_{e}}{q_{m}}$ **(1)**

$R_{L}=\frac{1}{1+K_{L1}C_{0}}$ **(2)**

$\ln q_{e}= lnk_{f1}+\frac{1}{n1}\ln C_{e}$ **(3)**

$q_{e}=\frac{q_{m}K_{L2}C_{e}}{1+K_{L2}C_{e}}$  **(4)**

$q_{e}=K_{f2}C_{e}^{1/n2}$  **(5)**

ln q_e_ = ln q_m_ − kε^2^ **(6)**

Where, C_e_ (mg/L) is the initial concentration of the studied pollutant at equilibrium, q_e_ (mg/g) is the capacity of the adsorbent for pollutant concentration at equilibrium, q_m_ (mg/g) is the adsorption maximum amount, 1/n, K_L_, K_F_, and K_DR_ are the heterogeneity factor, Langmuir coefficient (L/mg), Freundlich constant ((mg^1−(1/n)^ L^1/n^)/g), and the Dubinin–Radushkevich constant, respectively. At the same time, ε is the adsorption potential, given by **Eq. (7)**.

$\varepsilon=\mathrm{RTln}\left. \left( 1 + \frac{1}{C_{e}} \right. \right)$ **(7)**

where R (8.314 J/mol K) is the gas constant, and T is the temperature in kelvin.

**S2. Kinetic Studies**

To estimate the adsorption rate-limiting step, kinetic investigations were carried out for the adsorption of ARS by SCB and of CV and MG by SCB@ARS, using both linear and nonlinear forms of the Pseudo-1st-Order and Pseudo-2nd-Order models, along with the Elovich and intraparticle diffusion (IPD) models, as expressed in Eqs. (8), (9), (10), (11), (12), and (13), respectively.

$\frac{1}{q_{t}}= \frac{K_{1}}{q_{e}}+\frac{1}{q_{e}}$  **(8)**

$\frac{t}{q_{t}}= \frac{1}{K_{2}q_{e}^{2}}+\frac{1}{q_{e}t}$ **(9)**

$q_{t}=q_{e} ( 1\boldsymbol{-}\boldsymbol{e}^{\boldsymbol{-kt}} )$ **(10)**

$q_{t}=\frac{K_{2}q_{e}^{2}t}{1+ \mathbf{q}_{\mathbf{e}}K_{2}t}$ **(11)**

$q_{t}=\frac{1}{\beta} ln ( \alpha\beta)+\frac{1}{\beta} ln( t)$ **(12)**

$q_{t}=K_{\mathrm{diff}} {\times t}^{\frac{1}{2}}+\text{C}$ **(13)**

q_e_ (mg/g) and q_t_ (mg/g) represent the adsorption efficiency at equilibrium and at a certain time t (min), respectively. α (mg/g min) is the initial sorption rate, while β (g/mg) is the desorption constant. The constant C represents the intercept and reflects the boundary layer effect. Additionally, K_1_, K_2_, and K_diff_ correspond to pseudo-1^st^, pseudo-2^nd^ order, and intra-particle diffusion constants, respectively.

**S3. Thermodynamic studies**

Three basic thermodynamic parameters were studied for the adsorption process of ARS by SCB and the adsorption of CV and MG by SCB@ARS: the Gibbs free energy of adsorption (${\Delta G}_{\mathrm{ads}}^{o}$), the enthalpy change (${\Delta H}_{\mathrm{ads}}^{o}$), and the entropy change (${\Delta S}_{\mathrm{ads}}^{o}$) [3]. These parameters for the adsorption process were determined by using **Eqs. (14)**, **(15), and (16).**

 $K_{c}=\frac{C_{\mathrm{ad}}}{C_{e}}$ **(14)**

${\Delta G}_{\mathrm{ads}}^{o}= -RTIn K_{C}$ **(15)**

$\ln K_{C}= \frac{{\Delta S}_{\mathrm{ads}}^{o}}{R}- \frac{{\Delta H}_{\mathrm{ads}}^{o}}{\mathrm{RT}}$ **(16)**

where K_c_ is the thermodynamic equilibrium constant, but C_ad_ and C_e_ are the pollutant concentration taken by the adsorbent material at equilibrium (mg/g) and the pollutant concentration at equilibrium (mg/L), respectively. While R represents the universal gas constant, which is equivalent to 8.314 J/mol K.

**S4. Error functional analysis**

To analyze the error distribution between calculated values based on theoretical model correlations and experimental data, a variety of error functions were used to evaluate the fit of kinetic and isotherm models. The chi-square statistic (χ^2^), mean square error (MSE), and sum of squares error (SSE), which are explained in **Eqs. (17), (18), and (19)**, respectively, were the three error functions that were utilized.

$\chi^{2}=\sum_{i=1}^{n} \frac{({q_{ei}exp-q_{ei} cal)}^{2}}{q_{ei} cal}$ **(17)**

$\mathrm{MSE}=\frac{1}{N_{exp}}\sum_{i=1}^{n} ({q_{ei}exp-q_{ei} cal)}^{2}$ **(18)**

$\mathrm{SSE}=\sum_{i=1}^{n} ({q_{ei}exp-q_{ei} cal)}^{2}$ **(19)**

where n is the number of included observations. The subscript cal refers to theoretically calculated data, while the exp subscript represents experimental data.

**Table S1.** Chemical structures and properties of the investigated dyes.

| **Dye** | **Ionic nature** | **Other names** | **Molecular formula** | **Molecular weight** | **Molecular structure** |
| --- | --- | --- | --- | --- | --- |
| Alizarin red sulfonate | Anionic | Alizarine Carmine, Alizarine Red S, and Alizarine S | C_14_H_7_NaO_7_S | 342.26 g/mol |  |
| Crystal Violet | Cationic | basic violet 3, gentian violet, and methyl violet 10B | C_25_H_30_N_3_Cl | 407.98 g/mol |  |
| Methyl Green | Cationic | Light Green and Basic Blue 20 | C_26_H_33_N_3_Cl_2_ | 458.50 g/mol |  |

Figure S1. Linear BET plot of N_2_ adsorption-desorption isotherm for (a) SCB and (b) SCB@ARS.

|  | |  |
| --- | --- | --- |
|  |  | |

**Figure S2.** **(a)** linear Langmuir, **(b)** linear Freundlich, **(c)** Dubinin–Radushkevich, and **(d)** nonlinear Langmuir and Freundlich isotherm models for ARS adsorption using SCB.

| **** | **** |
| --- | --- |
| 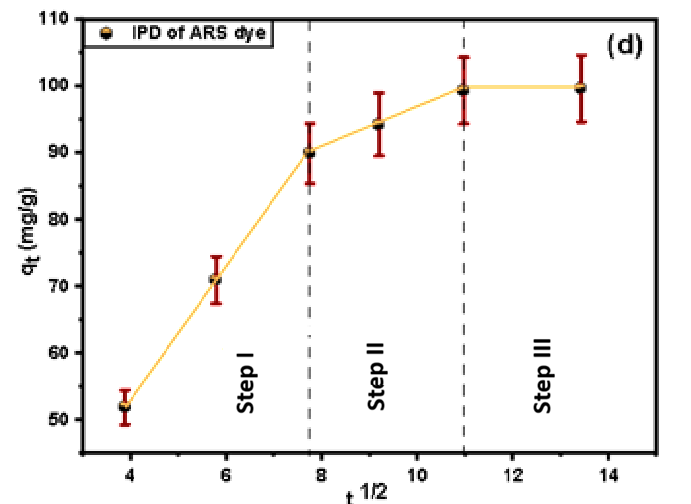 |  |
|  | |

**Figure S3.** **(a)** pseudo-1^st^-order, **(b)** pseudo-2^nd^-order, **(c)** IPD, **(d)** nonlinear pseudo-1^st^ and 2^nd^-order, and **(e)** Elovich kinetic models for ARS adsorption on SCB.

**Figure S4.** Plot of ln K_C_ versus (1/T) absolute temperature for the adsorption of ARS on the surface of pristine SCB adsorbent.

|  |  | |  |
| --- | --- | --- | --- |
|  | |  | |

**Figure S5.** Freundlich isotherm for **(a)** CV and **(b)** MG; Langmuir isotherm for **(c)** CV and **(d)** MG; D-R isotherm model for **(e)** CV and **(f)** MG; and nonlinear Langmuir and Freundlich isotherms for **(g)** CV and **(h)** MG.

|  |  |
| --- | --- |
| 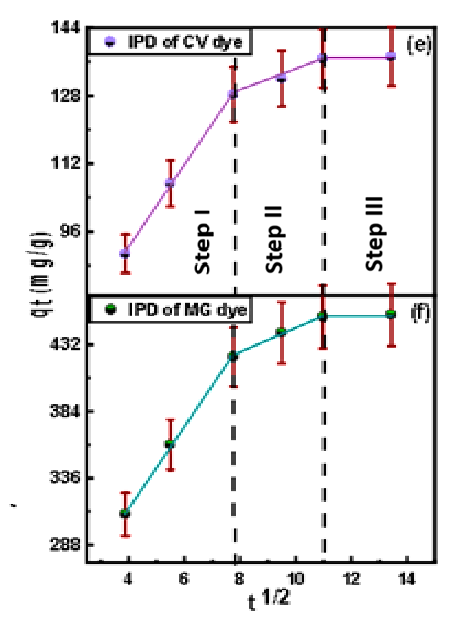 |  |
|  | |

**Figure S6.** pseudo-1^st^-order for **(a)** CV and **(b)** MG adsorption; pseudo-2^nd^-order for **(c)** CV and **(d)** MG adsorption; IPD for **(e)** CV and **(f)** MG adsorption; nonlinear pseudo-1^st^- and -2^nd^-order for **(g)** CV, and **(h)** MG adsorption; Elovich of **(i)** CV and **(j)** MG.

**Figure S7.** Plot of ln K_C_ versus (1/T) absolute temperature for the adsorption of **(a)** CV and **(b)** MG on the surface of SCB@ARS adsorbent (conditions: 10 mL aqueous solution of 150 mg/L of CV and 250 mg/L of MG and 0.005 g of SCB@ARS for 120 min at pH 10).

**References**

1. Nguyen, K. T., Ahmed, M. B., Mojiri, A., Huang, Y., Zhou, J. L., & Li, D. (2021). Advances in as contamination and adsorption in soil for effective management. Journal of Environmental Management, 296, 113274.
2. Dabhade, M. A., Saidutta, M. B., & Murthy, D. V. R. (2009). Adsorption of phenol on granular activated carbon from nutrient medium: equilibrium and kinetic study.
3. Rahman, M. A., Lamb, D., Kunhikrishnan, A., & Rahman, M. M. (2021). Kinetics, isotherms and adsorption–Desorption behavior of phosphorus from aqueous solution using zirconium–iron and iron modified biosolid biochars. Water, 13(23), 3320.
